# Conformation-Independent QSPR Approach for the Soil Sorption Coefficient of Heterogeneous Compounds

**DOI:** 10.3390/ijms17081247

**Published:** 2016-08-03

**Authors:** José F. Aranda, Juan C. Garro Martinez, Eduardo A. Castro, Pablo R. Duchowicz

**Affiliations:** 1 Instituto de Investigaciones Fisicoquímicas Teóricas y Aplicadas (INIFTA), CONICET, UNLP, Diag. 113 y 64, Sucursal 4, C.C. 16, La Plata 1900, Argentina; jfaranda10@gmail.com (J.F.A.); eacast@gmail.com (E.A.C.); 2 Instituto Multidisciplinario de Investigaciones Biológicas IMIBIO-SL (CCT San Luis), Departamento de Química, Universidad Nacional de San Luis, Chacabuco 917, San Luis 5700, Argentina; jcgarro@unsl.edu.ar

**Keywords:** Quantitative Structure-Property Relationships, Replacement Method, soil sorption coefficient, Pharmaceutical Data Exploration Laboratory software, Estimation Program Interface Suite software, Correlation and Logic software

## Abstract

We predict the soil sorption coefficient for a heterogeneous set of 643 organic non-ionic compounds by means of Quantitative Structure-Property Relationships (QSPR). A conformation-independent representation of the chemical structure is established. The 17,538 molecular descriptors derived with PaDEL and EPI Suite softwares are simultaneously analyzed through linear regressions obtained with the Replacement Method variable subset selection technique. The best predictive three-descriptors QSPR is developed on a reduced training set of 93 chemicals, having an acceptable predictive capability on 550 test set compounds. We also establish a model with a single optimal descriptor derived from CORAL freeware. The present approach compares fairly well with a previously reported one that uses Dragon descriptors.

## 1. Introduction

The soil sorption coefficient ($K_{oc}$) describes the biodegradation and pollution impact of organic pesticides [1] when these compounds interact with the organic matter of soils and sediments either on surface, ground or drinking water [2]. The reliable estimation of the $K_{oc}$ parameter is very important in agriculture, as its experimental measurement is difficult, expensive and time-consuming. Predicting the soil sorption coefficient for a wide number of chemical structures is very convenient in risk assessment [3].

In the realm of the Quantitative Structure-Property Relationships (QSPR) theory [4,5,6], an experimental property of a chemical compound, i.e., $K_{oc}$, can be predicted through the knowledge of its chemical structure. The structure is quantified by means of a set of suitable molecular descriptors, in other words, numerical quantities carrying specific information on the constitutional, topological, geometrical, hydrophobic, and/or electronic aspects [7,8,9]. Therefore, a set of descriptors is then statistically correlated with the experimental property, resulting in a mathematical model that can be used with find out useful parallelisms.

It is known that many published QSPR models that predict the soil sorption coefficient involve the experimental octanol/water partition coefficient ($K_{ow}$) or the water solubility ($S_{w}$) [10], while other QSPR are based on theoretical molecular descriptors [11,12,13]. However, usually, little work is done to examine the model’s predictivity (validation) and the chemical domain of application over a wide range of compounds, especially for new chemicals [14,15,16].

A previous QSPR study of Gramatica et al. [14] on a highly heterogeneous set of 643 organic non-ionic compounds predicts the soil sorption coefficient expressed in logarithmic units ($\log K_{oc}$). The training set with 93 compounds used in such work is peculiar, because it is much smaller than the test set of 550 compounds (1:6 ratio). The best Dragon molecular descriptors are selected through the Genetic Algorithms (GA) technique based on Multivariable Linear Regression analysis (MLR), leading to a four-dimensional QSPR having a predictivity of 78% on the test set. The best predicted data are obtained by consensus modeling from ten different models in the GA model population.

In this work, we report new alternative QSPR models for the soil sorption coefficient in the same molecular set studied by Gramatica et al. [14], using an approach that does not consider the conformational representation of the chemical structure by only relying on the constitutional and topological aspects of the molecules [15]. As is known, every model that includes three-dimensional descriptors usually involves high computational costs and long times during the calculation of molecular geometry optimization. Therefore, the conformation-independent QSPR approach can be considered as a very useful methodology.

In addition, we also explore the performance of QSPR models based on optimal descriptors [16]. Within this technique, the calculated optimal descriptor depends both on the molecular structure and the property under analysis ($K_{oc}$), but does not explicitly depend on the 3D-molecular geometry. We have shown the importance of optimal descriptors in previous QSPR studies [17,18,19,20,21].

## 2. Results and Discussion

We begin our QSPR analysis by exploring the performance of molecular descriptors calculated with the PaDEL freeware. The most representative structural features of the training set of 93 heterogeneous compounds are searched through the RM technique. In this way, the best MLR models based on 1–6 molecular descriptors are found in a pool having 17,536 variables. In order to remove the ‘collinear’ (identical) descriptors, the linearly-dependent pairs are identified within RM, and only one variable from each pair is kept for further analysis. This process leads to a set containing 3491 linearly-independent descriptors.

We follow the common practice of keeping the model’s dimension (*d*) as small as possible. The best MLR models are listed in Table 1, while a brief description of the descriptors meanings is provided in Table S1. It is appreciated from Table 1 that the $RMS_{\text{train}}$ parameter continues improving beyond four descriptors, but $RMS_{\text{test}}$ does not significantly improve. According to this, we choose a structure-property relationship having four descriptors with an acceptable predictive power on the test set:

(1) $$\log K_{oc}=0.18SP3+0.30CrippenLogP-0.090g\text{max}+0.16XLogP+1.18$$

$$N_{\text{train}}=93,\;R_{\text{train}}^{2}=0.87,\;RMS_{\text{train}}=0.45$$

$$R_{ij\text{max}}^{2}=0.58,\;o2.5=0,\;R_{\text{loo}}^{2}=0.85,\;RMS_{\text{LOO}}=0.47,\;RMS^{\text{rand}}=1.02$$

$$N_{\text{test}}=550,\;R_{\text{test}}^{2}=0.81,\;RMS_{\text{test}}=0.53$$

In this equation N is the number of compounds; $R_{ij\max}$ denotes the maximum correlation coefficient between descriptor pairs; o2.5 indicates the number of outlier compounds in the training set having a residual (difference between experimental and calculated activity) greater than 2.5-times $RMS_{\text{train}}$.

The conformation-independent descriptors appearing in Equation (1) belong to four different classes [9]: (i) a PaDEL Chi Path Descriptor: *SP3*, simple path of order 3; (ii) a Crippen descriptor: *CrippenLogP*, Crippen’s LogP; (iii) an electrotopological state atom type descriptor: *gmax*, the maximum E-state; and (iv) the *XLogP* descriptor.

**Figure 1:**
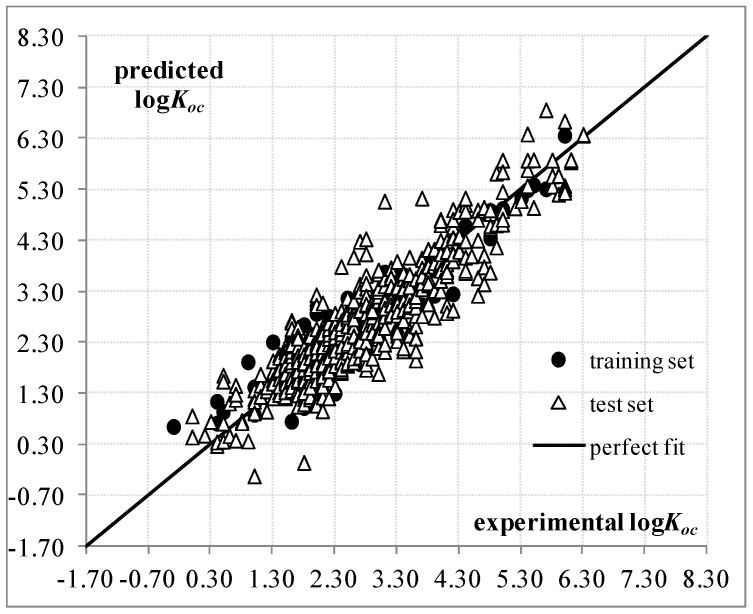
Predicted and experimental $\log K_{oc}$ values according to QSPR based on Equation (1).

A plot for the predicted $\log K_{oc}$ as a function of the experimental values for the training and test sets is provided in Figure 1. The dispersion plot of residuals in Figure S1 tends to obey a random pattern around the zero line, suggesting that the assumption of the MLR technique is fulfilled. The correlation matrix for Equation (1) is given in Table S2, showing the absence of high correlations between descriptor pairs, while their numerical values are included in Table S3.

Equation (1) has an acceptable predictive power on the external test set of 550 compounds, according to the $R_{\text{test}}^{2}$ and $RMS_{\text{test}}$ parameters. Such a model approves the internal validation process of Cross-Validation through the exclusion of one molecule at a time. The Y-Randomization technique demonstrates that Equation (1) has $RMS_{\text{train}}<RMS^{\text{rand}}$ and thus a valid structure-$\log K_{oc}$ relationship is found. The external validation criteria recommended in [22] to assure predictive capability are also achieved and are summarized in Table S4.

The statistical quality of Equation (1) is quite similar to various QSPR models reported previously by Gramatica et al. [14]. For instance, our QSPR with $RMS_{\text{train}}=0.45$ and $RMS_{\text{test}}=0.53$ is better than the published four-topological descriptor model with $RMS_{\text{train}}=0.52$ and $RMS_{\text{test}}=0.56$. Furthermore, Equation (1) is also comparable to the three-descriptor consensus model proposed in that paper ($RMS_{\text{train}}=0.52$ and $RMS_{\text{test}}=0.53$), although such a model has as the disadvantage that it includes geometrical descriptors. In our approach, we do not consider the geometrical representation of the chemical structures, but consider their constitutional and topological aspects instead while achieving acceptable results.

As a next step of this QSPR study, we include optimal molecular descriptor definitions in order to analyze the performance of such soil sorption-specific structural variables. The DCW optimal descriptor is optimized by increasing $R_{\text{train}}^{2}$, until the model starts to lose predictive capability in the test set (measured by $RMS_{\text{test}}$). The best structural representation for the 93 training compounds is hydrogen-filled graph, where the statistics for the stepwise evolution of the linear model is presented in Table 2. The first local descriptor selected is NNC (Nearest Neighboring Code), then the following ones are ${}^{0}EC$ (Morgan Extended Connectivity of zero-th order) and NOSP (the presence of Nitrogen, Oxygen, Sulfur or Phosphorus) in that order. It is noted from Table 2 that the best quality optimal descriptor involves such three-variable types, and 64 active attributes are based on them (shown in Table S5). More complete details for the QSPR model are the following:

(2) $$\log K_{oc}=0.073DCW+0.31$$

$$N_{\text{train}}=93,\;R_{\text{train}}^{2}=0.87,\;RMS_{\text{train}}=\;0.45$$

$$o2.5=1,\;R_{\text{loo}}^{2}=0.86,\;RMS_{\text{LOO}}=0.45,\;RMS^{\text{rand}}=1.11$$

$$N_{\text{test}}=550,\;R_{\text{test}}^{2}=0.76,\;RMS_{\text{test}}=0.61$$

The parameters used for the DCW calculation are T=1 and $N_{\text{epochs}}=7$. Figures S2 and S3 demonstrate that the MLR technique is also satisfied for Equation (2). An example for the calculation of DCW for formaldehyde is provided in Table 3.

Our results reveal that Equation (1) has a better performance on the test set than Equation (2). Both QSPRs are obtained through different approaches, i.e., by allowing or not the molecular descriptor representing the chemical structure to be dependent on the studied $\log K_{oc}$ property.

As a next step, we investigate what happens when the previous set of 3491 0D–2D descriptors from PaDEL is combined with the optimal DCW descriptor. The best 1–6 variable MLR models found in such pool of 3492 descriptors (Table S6) do not ameliorate the predictive power of our first model, as the training set statistics is better but not the one for the test set.

In a new attempt to improve Equation (1), we consider the inclusion of EPI Suite predictions as semiempirical molecular descriptors, calculated through $\log K_{ow}Epi$ and $\log S_{w}Epi$ predicted values. After searching the best MLR models in the set composed of 3493 independent descriptors from PaDEL and EPI Suite (refer to Table 4), the following structure-$K_{oc}$ relationship is achieved:

(3) $$\log K_{oc}=0.60MLFER.E-0.36SubFP302+0.48\log K_{ow}Epi+0.72$$

$$N_{\text{train}}=93,\;R_{\text{train}}^{2}=0.87,\;RMS_{\text{train}}=\;0.44$$

$$R_{ij\;\text{max}}^{2}=0.21,\;o2.5=0,\;R_{\text{loo}}^{2}=0.86,\;RMS_{\text{LOO}}=0.46,\;RMS^{\text{rand}}=1.02$$

$$N_{\text{test}}=550,\;R_{\text{test}}^{2}=0.84,\;RMS_{\text{test}}=0.48$$

**Figure 2:**
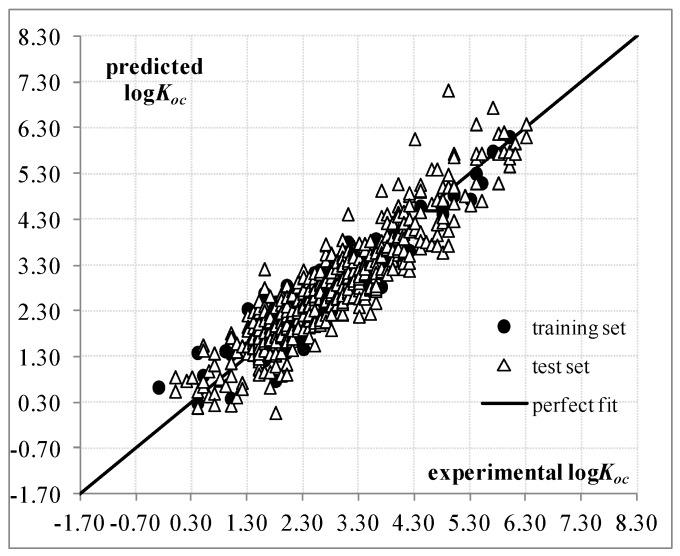
Predicted and experimental $\log K_{oc}$ values according to QSPR based on Equation (3).

The performance of Equation (3) is better than Equation (1), and thus, we consider that this new QSPR model is the most suitable structure-soil sorption coefficient relationship for the 643 organic non-ionic compounds. Figure 2 and Figure S4 plot the predictions, while Tables S2 and S4 provide the correlation matrix and external validation criteria for Equation (3).

The 2D molecular descriptors appearing in this last equation belong to three different classes: (i) a Molecular Linear Free Energy Relation (MLFER) descriptor: *MLFER.E*, measuring the excessive molar refraction; (ii) a substructure fingerprint: SubFP302, the presence of rotatable bonds; and (iii) an EPI Suite descriptor: $\log K_{ow}Epi$. As the three descriptors take positive numerical values, Equation (3) indicates that a compound having higher values for both *MLFER.E* and $\log K_{ow}Epi$ descriptors together with a lower value for *SubFP302* tend to have a higher predicted soil sorption coefficient.

*MLFER.E* measures the excessive molar refraction: the molar refraction of the solute minus the molar refraction of an alkane of equivalent volume. This descriptor can be easily estimated from the knowledge of a compound’s refractive index, and suggests the propensity of the soil phase to interact with solute compounds having π- and σ-electron pairs.

The *SubFP302* descriptor has a clear interpretation as quantifies the presence (equal to one) or absence (equal to zero) of rotatable bonds in the chemical structure. This fingerprint identifies rotatable bonds that allow free rotation around themselves, that is to say, any single bond, not in a ring, bound to a non-terminal heavy atom.

Finally, the logarithm of the octanol/water partition coefficient $\log K_{ow}Epi$ descriptor is a well-known physicochemical property that has been widely used in past QSPR studies for correlating the $\log K_{oc}$ values. Therefore, hydrophobic compounds with high $\log K_{ow}Epi$ values tend to exhibit a higher retaining by the organic matter of soils and sediments.

The analysis of the applicability domain of the new proposed QSPR reveals that 16 compounds out of the 550 included in the test set do not belong to the AD of the model, as $h_{i}>h^{*}=0.13$. The obtained leverage values are also provided in Table S7. We assume that this particular behavior is due to the complexity of the dataset, i.e., the great structural heterogeneity of the molecules considered in this study. Thus, the predicted $\log K_{oc}$ values for all, with the exception to such 16 test set compounds, can be considered as reliable as they fall within the AD.

As a final comparison, our best QSPR model with $RMS_{\text{train}}=0.44$ and $RMS_{\text{test}}=0.48$ has a better performance on the heterogeneous compounds than the one provided by EPI Suite: $RMS_{\text{train}}=0.47$ and $RMS_{\text{test}}=0.56$ (connectivity method) and $RMS_{\text{train}}=0.48$ and $RMS_{\text{test}}=0.56$ (partition coefficient based method). This means that our developed QSPR model of Equation (3) represents an alternative/complementary tool to the EPI Suite program for predicting the studied property in present dataset of 643 organic non-ionic compounds.

## 3. Materials and Methods

### 3.1. Experimental Dataset

The experimental soil sorption partition coefficient collected from [14] is quantified as the ratio between chemical concentration in soil and in water normalized to organic carbon. In the present dataset, $\log K_{oc}$ ranges in the interval (−0.31, 6.02) in the training set (train) and (0, 6.33) in the test set (test); the complete list of 643 compounds studied here is included in Table S7 as Supplementary Material. The dataset is highly heterogeneous, and includes practically all of the principal functional groups present in pesticides and various organic pollutants.

In addition and for comparison purposes, the calculated logarithm of the soil sorption partition coefficient is obtained through the Estimation Program Interface (EPI Suite) software from the KOCWIN module ($\log K_{oc}Epi$) [23]. EPI Suite calculates $\log K_{oc}Epi$ via two different techniques: (a) based on the first order Molecular Connectivity Index (MCI); and (b) based on $\log K_{ow}$ (rather than MCI). In both cases, the program employs a series of group contribution factors.

### 3.2. Structural Representation and Molecular Descriptors Calculation

The molecules are first drawn in mol format with ACDLabs ChemSketch freeware [24]. The set of conformation-independent molecular descriptors is computed using PaDEL Version 2.20 [25], because it has the advantage that it is a freely available and open source software. PaDEL currently calculates 1444 0D–2D descriptors and 12 fingerprint types (total 16,092 bits) [26]. Furthermore, semiempirical descriptors from EPI Suite are added, such as the calculated logarithm of the octanol/water partition coefficient from KOWWIN ($\log K_{ow}Epi$) and the calculated logarithm of the water solubility from WATERNT ($\log S_{w}Epi$) [23].

Therefore, the total number of non-conformational descriptors explored in this work is 17,538. It is our intention to capture, with such a great number of descriptors, the most relevant structural characteristics affecting the studied property.

### 3.3. Model Development

#### 3.3.1. Molecular Descriptors’ Selection in Multivariable Linear Regression (MLR)

We employ the Replacement Method (RM) technique [27,28,29,30,31,32,33] in order to generate MLR models on the training set, by searching in a pool having D=17,538 descriptors for optimal subsets containing *d* descriptors (*d* is much lower than *D*), with smallest values for the standard deviation ($S_{\text{train}}$) or the root mean square deviation ($RMS_{\text{train}}$). Table S8 includes a list of mathematical equations involved in the present study. All of the MATLAB-programmed [34] algorithms used in our calculations are available upon request.

#### 3.3.2. The Optimal Molecular Descriptors

By means of the CORAL freeware (Correlation and Logic) [35] it is easy to define different optimal molecular descriptors. The Structural Representation (SR) used, i.e., graph or SMILES (Simplified Molecular Input Line Entry Specification), determines the Structural Attributes or local descriptors (SA) available for the QSPR. Therefore, it is necessary to decide which SA combination is the most appropriate, and this is done in a stepwise fashion, i.e., first search for the best single SA, then search for a second SA that combines the best with the previous one, and so on.

The *DCW* descriptor is a linear combination of Correlation Weights (*CW*); refer to Table S8. The *CW* is calculated for each SA in the training set through the Monte Carlo (MC) simulation method. The *DCW* depends on the threshold (*T*) and the number of epochs ($N_{\text{epochs}}$): the appropriate selection of *T* and $N_{\text{epochs}}$ avoids model over-fitting. The rare attributes are the ones that occur in less than *T* compounds, and in this work *T* is a positive integer analyzed in the range from 0-5.

#### 3.3.3. Model Validation

The linear regression models are theoretically validated through Leave-One-Out Cross-Validation (LOO) [22]. A more reliable validation is applied with an external test set of structures: the same training set-test set partition from [14] is used in present analysis, that is to say, 93 compounds in the training set and 550 compounds in the test set. We also scramble the experimental property values with Y-Randomization [36] and 10,000 cases, as a way of checking that the model is not a result of chance correlation when $RMS^{\text{rand}}$ is greater than $RMS_{\text{train}}$.

#### 3.3.4. Applicability Domain

A predictive QSPR model is only able to predict molecules falling within its Applicability Domain (AD) [37], so that the predicted property is not a result of substantial extrapolation (unreliable prediction). The AD definition is dependent on the model’s descriptors and the experimental property. Within the leverage approach [38], a test set compound must have a calculated leverage ($h_{i}$) smaller than the warning leverage ($h^{*}$).

## 4. Conclusions

We have succeeded in establishing structure-property relationships for the soil sorption coefficient, a useful parameter related to sorption processes determining the environmental fate, distribution and persistence of chemicals. The chemical domain explored includes a heterogeneous set of 643 organic non-ionic compounds, having a $K_{oc}$ range of more than six log units. The QSPR models found on a training set composed of 93 compounds have an acceptable predictive performance on a test set including 550 compounds, and are able to fulfill other necessary mathematical conditions, such as Cross-Validation, Y-Randomization and Applicability Domain analysis. Our results compare favorably to previous reported ones from the literature, although the proposed models involve molecular descriptors calculated through freely available software like PaDEL, CORAL and EPI Suite.

As we have developed a conformation-independent QSPR approach, the conformational representation of the chemical structures is avoided, and thus, no-experimental information on the X-ray crystal structure of compounds is required. Our research work continuously focuses on the use of new methods based on constitutional and topological approximations to QSPR studies, and thus, new results will be published shortly elsewhere.

## Figures and Tables

**Table 1:** The best linear QSPR models obtained from a pool of 3491 geometry independent descriptors obtained from PaDEL freeware; the selected model appears in bold.

*d*	Descriptors	$R_{\text{train}}^{2}$	$R_{\text{test}}^{2}$	$RMS_{\text{train}}$	$RMS_{\text{test}}$
1	*CrippenLogP*	0.72	0.68	0.65	0.67
2	*CrippenLogP XLogP*	0.80	0.76	0.55	0.59
3	*CrippenLogP gmax TpiPC*	0.84	0.79	0.49	0.56
**4**	***SP3 CrippenLogP gmax XLogP***	**0.87**	**0.81**	**0.45**	**0.53**
5	*ALogp2 CrippenLogP maxHBint2 TpiPC XLogP*	0.87	0.81	0.44	0.52
6	*BCUTw-1l CrippenLogP gmax ETA_Epsilon_3 WPOL XLogP*	0.89	0.81	0.41	0.53

**Table 2:** The stepwise search for finding the best structural attributes contributing the optimal descriptor; the selected result appears in bold. NNC, Nearest Neighboring Code; ${}^{0}EC$, Morgan Extended Connectivity of zero-th order; NOSP, the presence of Nitrogen, Oxygen, Sulfur or Phosphorus.

Structural Attributes	$R_{\text{train}}^{2}$	$R_{\text{test}}^{2}$	${\mathrm{RMS}}_{\text{train}}$	${\mathrm{RMS}}_{\text{test}}$	$N_{\text{act}}$
NNC	0.84	0.73	0.49	0.64	50
$NNC\;{}^{0}EC$	0.86	0.75	0.46	0.62	70
$NNC\;{}^{0}EC\;NOSP$	**0.87**	**0.76**	**0.45**	**0.61**	**64**

**Table 3:** An example of the calculation of the optimal descriptor for formaldehyde by summing *CW* values: DCW=−0.64892.

Structural Attribute	*CW*
EC0-O...1...	0.12508
EC0-C...3...	1.00094
EC0-H...1...	−0.18254
EC0-H...1...	−0.18254
NNC-O...101.	0.24867
NNC-C...303.	−0.75284
NNC-H...101.	−0.07978
NNC-H...101.	−0.07978
NOSP01000000	−0.74613

**Table 4:** The best linear QSPR models obtained from a pool of 3493 geometry independent descriptors obtained from PaDEL and EPI Suite softwares; the selected model appears in bold.

*d*	Descriptors	$R_{\text{train}}^{2}$	$R_{\text{test}}^{2}$	${\mathrm{RMS}}_{\text{train}}$	${\mathrm{RMS}}_{\text{test}}$
1	$\log K_{ow}Epi$	0.77	0.76	0.59	0.59
2	*MLFER_E* $\log K_{ow}Epi$	0.86	0.83	0.46	0.50
**3**	***MLFER_E SubFP302*** $\log K_{ow}Epi$	**0.87**	**0.84**	**0.44**	**0.48**
4	*mindO MLFER_E KRFP1105* $\log K_{ow}Epi$	0.88	0.84	0.42	0.48
5	*MAXDP2 ZMIC1 TpiPC KRFP3788* $\log K_{ow}Epi$	0.90	0.84	0.40	0.49
6	*ATSC3c AATSC3c MATS4p MLFER_E AD2D393* $\log K_{ow}Epi$	0.91	0.84	0.37	0.49

## Supplementary Materials

Supplementary materials can be found at http://www.mdpi.com/1422-0067/17/8/1247/s1.

## References

[1] Sparks D.L. (2013). Environmental Soil Chemistry.

[2] Jury W.A., Henn S.C., Melancon S.M. (1986). Adsorption of organic chemicals onto soil. Vadose Zone Modeling of Organic Pollutants.

[3] Gawlik B.M., Sotiriou N., Feicht E.A., Schulte-Hostede S., Kettrup A. (1997). Alternatives for the determination of the soil adsorption coefficient, KOC, of non-ionic organic compounds—A review. Chemosphere.

[4] Hansch C., Leo A. (1995). Exploring QSAR. Fundamentals and Applications in Chemistry and Biology.

[5] Kubinyi H. (2008). QSAR: Hansch Analysis and Related Approaches.

[6] Puzyn T., Leszczynski J., Cronin M.T.D. (2010). Recent Advances in QSAR Studies: Methods and Applications.

[7] Katritzky A.R., Goordeva E.V. (1993). Traditional topological indices vs. Electronic, geometrical, and combined molecular descriptors in QSAR/QSPR research. J. Chem. Inf. Comput. Sci..

[8] Diudea M.V.E. (2001). QSPR/QSAR Studies by Molecular Descriptors.

[9] Todeschini R., Consonni V. (2009). Molecular Descriptors for Chemoinformatics (Methods and Principles in Medicinal Chemistry).

[10] Sabljic A., Gusten H., Verhaar H., Hermens J. (1995). QSAR modeling of soil sorption. Improvements and systematics of log Koc vs. Log kow correlations. Chemosphere.

[11] Duchowicz P.R., González M.P., Helguera A.M., Cordeiro M.N.D.S., Castro E.A. (2007). Application of the replacement method as novel variable selection in QSPR. 2. Soil sorption coefficients. Chemom. Intell. Lab. Syst..

[12] Goudarzi N., Goodarzi M., Araujo M.C.U., Galvão R.K.H. (2009). QSPR modeling of soil sorption coefficients (*K_oc_*) of pesticides using SPA-ANN and SPA-MLR. J. Agric. Food Chem..

[13] Shao Y., Liu J., Wanga M., Shi L., Yao X., Gramatica P. (2014). Integrated QSPR models to predict the soil sorption coefficient for a large diverse set of compounds by using different modeling methods. Atmos. Environ..

[14] Gramatica P., Giani E., Papa E. (2007). Statistical external validation and consensus modeling: A QSPR case study for Koc prediction. J. Mol. Graph. Model..

[15] Duchowicz P.R., Comelli N.C., Ortiz E.V., Castro E.A. (2012). QSAR study for carcinogenicity in a large set of organic compounds. Curr. Drug Saf..

[16] Toropov A.A., Toropova A.P., Benfenati E., Gini G. (2013). OCWLGI descriptors: Theory and praxis. Curr. Comput. Aided Drug Des..

[17] Ibezim E., Duchowicz P.R., Ortiz E.V., Castro E.A. (2012). QSAR on aryl-piperazine derivatives with activity on malaria. Chemom. Intell. Lab. Syst..

[18] Mullen L.M.A., Duchowicz P.R., Castro E.A. (2011). QSAR treatment on a new class of triphenylmethyl-containing compounds as potent anticancer agents. Chemom. Intell. Lab. Syst..

[19] Toropov A.A., Leszczynska D., Leszczynski J. (2007). Predicting water solubility and octanol water partition coefficient for carbon nanotubes based on the chiral vector. Comput. Biol. Chem..

[20] Toropov A.A., Toropova A.P., Benfenati E., Gini G., Puzyn T., Leszczynska D., Leszczynski J. (2012). Novel application of the CORAL software to model cytotoxicity of metal oxide nanoparticles to bacteria *Escherichia coli*. Chemosphere.

[21] Toropova A.P., Toropov A.A., Martyanov S.E., Benfenati E., Gini G., Leszczynska D., Leszczynski J. (2012). CORAL: QSAR modeling of toxicity of organic chemicals towards Daphnia magna. Chemom. Intell. Lab. Syst..

[22] Golbraikh A., Tropsha A. (2002). Beware of q^2^!. J. Mol. Graph. Model..

[23] US EPA. https://www.epa.gov/tsca-screening-tools/epi-suitetm-estimation-program-interface.

[24] ACD/ChemSketch, 2016. http://www.acdlabs.com.

[25] PaDEL, 2016. http://www.yapcwsoft.com/.

[26] Yap C.W. (2011). PaDEL-descriptor: An open source software to calculate molecular descriptors and fingerprints. J. Comput. Chem..

[27] Duchowicz P.R., Castro E.A., Fernández F.M. (2006). Alternative algorithm for the search of an optimal set of descriptors in QSAR-QSPR studies. MATCH Commun. Math. Comput. Chem..

[28] Duchowicz P.R., Castro E.A., Fernández F.M., González M. (2005). A new search algorithm of QSPR/QSAR theories: Normal boiling points of some organic molecules. Chem. Phys. Lett..

[29] Duchowicz P.R., Talevi A., Bruno-Blanch L.E., Castro E.A. (2008). New QSPR study for the prediction of aqueous solubility of drug-like compounds. Bioorg. Med. Chem. Lett..

[30] Goodarzi M., Duchowicz P.R., Wu C.H., Fernández F.M., Castro E.A. (2009). New hybrid genetic based support vector regression as QSAR approach for analyzing flavonoids-GABA(A) complexes. J. Chem. Inf. Model..

[31] Pomilio A.B., Giraudo M.A., Duchowicz P.R., Castro E.A. (2010). QSPR analyses for aminograms in food: Citrus juices and concentrates. Food. Chem..

[32] Talevi A., Goodarzi M., Ortiz E.V., Duchowicz P.R., Bellera C.L., Pesce G., Castro E.A., Bruno-Blanch L.E. (2011). Prediction of drug intestinal absorption by new linear and non-linear QSPR. Eur. J. Med. Chem..

[33] Pasquale G., Romanelli G.P., Autino J.C., García J., Ortiz E.V., Duchowicz P.R. (2012). Quantitative structure-activity relationships on chalcone derivatives: Mosquito larvicidal studies. J. Agric. Food. Chem..

[34] Matlab 7.0. http://www.mathworks.com.

[35] Coral 1.5. http://www.insilico.eu/coral.

[36] Wold S., Eriksson L., van de Waterbeemd H. (1995). Statistical validation of qsar results. Chemometrics Methods in Molecular Design.

[37] Gramatica P. (2007). Principles of qsar models validation: Internal and external. QSAR Comb. Sci..

[38] Eriksson L., Jaworska J., Worth A.P., Cronin M.T., McDowell R.M., Gramatica P. (2003). Methods for reliability and uncertainty assessment and for applicability evaluations of classification- and regression-based QSARS. Environ. Health Perspect..

