# Supplementary material for: Conformation-Independent QSPR Approach for the Soil Sorption Coefficient of Heterogeneous Compounds
Source: Int J Mol Sci. 2016 Aug 3;17(8):1247. doi: 10.3390/ijms17081247 (PMC5000645; doi:10.3390/ijms17081247)
Supplement: Supplementary file 1 [file ijms-17-01247-s001.pdf]

Supplementary Materials: Conformation-Independent QSPR Approach for the Soil Sorption Coefficient of Heterogeneous Compounds

José F. Aranda, Juan C. Garro Martinez, Eduardo A. Castro and Pablo R. Duchowicz

Table S1. A brief description for different molecular descriptors.

Class	Symbol	Brief Description
Crippen descriptor	*CrippenLogP*	Crippen's LogP
XLogP descriptor	*XLogP*	XLogP
ALOGP descriptor	*ALogp2*	Square of *ALogP* (Ghose-Crippen $\log K_{ow}$)
EPI Suite descriptor	$\log K_{ow}Epi$	EPI Suite calculated logarithm of octanol/water partition coefficient
	$\log S_{w}Epi$	EPI Suite calculated logarithm of water solubility
Path count descriptor	*TpiPC*	Total conventional bond order (up to order 10) (ln(1 + *x*))
BCUT descriptor	*BCUTw-1l*	nhigh lowest atom weighted BCUT
Electrotopological State Atom Type descriptor	*gmax*	Maximum E-State
	maxHBint2	Maximum E-State descriptor of strength for potential hydrogen bonds of path length 2
	mindO	Minimum atom-type E-State: =O
	MAXDP2	Maximum positive intrinsic state difference in the molecule (related to the electrophilicity of the molecule). Using deltaV = Zv-maxBondedHydrogens
Structural Attribute Type descriptor	*NNC*	Nearest neighboring code of hydrogen filled graph
	^{0}EC	Morgan extended connectivity of zero-th order of hydrogen filled graph
	NOSP	Presence of nitrogen, oxygen, sulfur or phosphorus of SMILES
Optimal descriptor	*DCW*	Optimal descriptor based on NNC, ^{0}EC and $NOSP$ attributes types
PaDEL Chi Path descriptor	*SP3*	Simple path, order 3
Extended Topochemical Atom descriptor	*ETA_Epsilon_3*	A measure of electronegative atom count
Wiener Numbers descriptor	*WPOL*	Weiner polarity number
MLFER descriptor	*MLFER.E*	Excessive molar refraction
Substructure Fingerprint	*SubFP302*	Rotatable bond, SMARTS: [!$(*#*)&!D1]-!@[!$(*#*)&!D1]
Information Content descriptor	*ZMIC1*	Z-modified information content index (neighborhood symmetry of 1-order)

Table 1. *Cont.*

Class	Symbol	Brief Description
Autocorrelation descriptor	*ATSC3c*	Centered Broto-Moreau autocorrelation—lag 3/weighted by charges
	AATSC3c	Average centered Broto-Moreau autocorrelation—lag 3/weighted by charges
	MATS4p	Moran autocorrelation—lag 4/weighted by polarizabilities
	MATS4i	Moran autocorrelation—lag 4/weighted by first ionization potential
2D Atom Pairs Fingerprint	*AD2D393*	Presence of C-O at topological distance 6
Klekota Roth Fingerprint	*KRFP1105*	Presence of SMARTS substructure: [!#1][NH]c1nc([!#1])nc([!#1])n1
	KRFP3788	Presence of SMARTS substructure: CCO
	KRFPC2306	Count of SMARTS substructure: [!#1]N([CH3])[CH3]
CDK Fingerprint	*FP541*	CDK fingerprint number 541
CDK Extended Fingerprint	*ExtFP394*	CDK extended fingerprint number 394

Table S2. Correlation matrices for Quantitative Structure-Property Relationships.

**Equation (1)**	***SP3***	***CrippenLogP***	***gmax***	***XlogP***
SP3	1.00	0.60	0.25	0.58
CrippenLogP		1.00	0.18	0.76
gmax			1.00	0.23
XLogP				1.00
**Equation (3)**	***MLFER.E***	***SubFP302***	$\log K_{ow}Epi$	
MLFER.E	1.00	0.05	0.21	
SubFP302		1.00	0.04	
$\log K_{ow}Epi$			1.00	

Table S3. Numerical values for the most relevant molecular descriptors involved in QSPR models.

ID	*SP3*	*CrippenLogP*	*gmax*	*XLogP*	*DCW*	*MLFER.E*	*SubFP302*	$\log K_{ow}Epi$
1	0.00000000	0.04899000	8.00000000	0.02100000	−0.80470000	0.16200000	0	0.35000000
2	6.13676261	6.47880000	2.39859284	7.02000000	72.21379000	1.85200000	1	6.79000000
3	8.62400783	5.73720000	2.30907407	10.21200000	75.33712000	3.46400000	0	6.11000000
4	3.34841462	2.48758000	10.62525943	1.59800000	21.10358000	1.01700000	1	1.18000000
5	3.95977831	1.51450000	11.80486883	0.52700000	23.85125000	0.67700000	1	0.42000000
6	9.21295342	6.29940000	2.33277778	11.56600000	80.31421000	3.60000000	0	6.70000000
7	4.18315398	1.50720000	4.13703704	1.48500000	28.83051000	0.95100000	1	1.00000000
8	2.59263896	0.75389000	10.42314815	1.93800000	19.29280000	0.99200000	1	0.74000000
9	5.46852057	2.99181000	5.97258755	4.07200000	39.72869000	1.60700000	1	4.08000000
10	0.00000000	2.55290000	1.36959877	2.86400000	29.87455000	0.42500000	0	2.44000000
11	5.32280178	3.36259000	10.54115403	4.32600000	31.84168000	1.54200000	1	3.73000000
12	9.15354793	5.27282000	2.39361111	10.31900000	75.93011000	3.09600000	0	7.05000000
13	6.82477710	4.90758000	9.41067969	5.66200000	49.15213000	1.78600000	1	5.64000000
14	7.27847215	5.14620000	2.29861111	9.69000000	65.09428000	2.86400000	0	5.52000000
15	0.00000000	−1.37821000	9.00000000	−1.68600000	2.06704000	0.44700000	0	−1.56000000
16	0.81649658	−0.64050000	8.11111111	−0.74800000	3.78314000	0.34100000	1	−0.78000000
17	8.33545694	5.20224000	2.27027778	10.33600000	73.32189000	2.88000000	0	6.62000000
18	4.97606774	3.64440000	1.35354595	3.83400000	51.58705000	0.99200000	0	4.26000000
19	4.19739619	4.37639000	9.63771262	2.21700000	34.16673000	1.16900000	0	4.09000000
20	4.84333659	3.47958000	5.56166703	4.85900000	40.64055000	1.68000000	1	3.19000000
21	5.54212870	3.96338000	4.18340892	5.48800000	45.19926000	1.65500000	1	4.29000000
22	2.42166830	1.22140000	8.51968821	3.18900000	17.16042000	0.82900000	1	1.57000000
23	3.64472044	0.88739000	10.97842834	0.89900000	17.84944000	1.23000000	1	0.72000000
24	10.32917914	4.40759000	6.10054744	3.00500000	59.01298000	1.88500000	0	5.45000000
25	1.64384685	−0.68981000	5.04861111	−0.38000000	11.13638000	1.21300000	0	−0.47000000
26	3.70211466	1.12039000	10.31222600	1.92000000	18.64219000	1.05700000	1	1.69000000
27	1.89384685	2.37709000	5.35777778	1.97700000	17.98879000	0.93100000	0	1.08000000
28	0.00000000	0.29240000	5.10596708	−0.24000000	11.45626000	0.77300000	0	−0.83000000

Table S3. *Cont.*

ID	*SP3*	*CrippenLogP*	*gmax*	*XLogP*	*DCW*	*MLFER.E*	*SubFP302*	$\log K_{ow}Epi$
29	0.00000000	−0.81130000	4.88374486	−0.80100000	10.18309000	0.96200000	0	−1.31000000
30	3.03234801	1.62659000	11.30369684	1.20700000	10.79018000	0.53000000	1	0.60000000
31	5.12524185	2.65678000	11.09156226	4.08900000	29.88534000	1.57800000	1	2.35000000
32	2.84973598	1.40330000	10.39111867	1.69800000	17.87191000	1.08200000	1	1.25000000
33	2.52948273	1.37528000	10.27981859	1.37500000	15.78834000	1.07400000	1	0.71000000
34	0.00000000	−0.00140000	7.56944444	−0.07600000	2.88166000	0.20500000	0	−0.14000000
35	0.00000000	0.32479000	9.00000000	−0.08000000	−0.64387000	0.15200000	0	0.09000000
36	2.59263896	1.35319000	10.20092593	2.66600000	15.30872000	0.77600000	1	1.87000000
37	0.00000000	−0.39150000	7.00000000	−0.49900000	0.79809000	0.20500000	0	−0.63000000
38	0.00000000	1.98640000	1.34876543	2.07400000	24.39846000	0.35500000	0	1.52000000
39	2.25000000	3.72680000	1.38022977	3.88200000	38.89110000	0.76600000	1	4.03000000
40	0.50000000	0.38870000	7.87500000	0.28200000	5.18337000	0.20500000	1	0.35000000
41	0.70710678	0.77880000	8.06625000	0.85100000	7.48508000	0.20500000	1	0.84000000
42	0.95710678	1.16890000	8.19736111	1.42000000	9.78678000	0.20500000	1	1.33000000
43	1.50000000	1.68660000	2.00000000	4.06200000	19.43450000	0.65600000	0	1.99000000
44	0.00000000	2.37650000	2.40560700	2.46900000	26.48204000	0.34900000	0	2.68000000
45	10.32917914	4.40759000	6.10054744	3.00500000	57.16892000	1.88500000	0	5.45000000
46	6.95325919	5.68999000	5.28466435	5.94000000	50.55728000	1.62600000	1	5.67000000
47	5.97948950	5.91230000	2.36721167	6.36700000	60.52482000	1.76500000	1	5.87000000
48	5.97948950	6.00519000	2.30934130	6.51500000	55.32218000	1.96400000	1	6.00000000
49	0.00000000	1.01110000	2.23379630	1.37800000	10.68381000	0.34000000	0	1.18000000
50	0.00000000	1.42150000	1.57716049	1.51900000	18.92238000	0.27700000	0	1.34000000
51	0.00000000	0.01660000	4.50000000	−0.19000000	3.40631000	0.26100000	0	−0.05000000
52	0.00000000	2.45470000	1.53472222	3.08800000	16.11102000	0.87400000	0	1.79000000
53	0.00000000	2.14250000	1.39429012	2.41200000	21.63598000	0.52800000	0	1.61000000
54	0.00000000	1.81000000	2.31207133	1.81600000	21.00595000	0.26200000	0	1.76000000
55	0.50000000	1.86139000	2.23628258	2.25800000	15.03971000	0.43000000	0	1.98000000
56	0.00000000	2.28360000	11.90560700	2.72700000	24.98924000	0.22200000	0	2.13000000
57	1.73205081	1.49869000	10.10262346	0.77200000	15.40604000	0.36800000	1	1.68000000

Table S3. *Cont.*

ID	*SP3*	*CrippenLogP*	*gmax*	*XLogP*	*DCW*	*MLFER.E*	*SubFP302*	$\log K_{ow}Epi$
58	1.73205081	1.44980000	9.94907407	1.60400000	22.48257000	0.56100000	1	1.32000000
59	8.77745261	5.09388000	2.73072188	3.55000000	51.06341000	1.76400000	0	5.86000000
60	4.75384552	4.40468000	1.99819959	3.69600000	36.35494000	1.27600000	0	4.63000000
61	4.15165043	6.69440000	13.24505763	5.35800000	46.70357000	1.18000000	1	5.75000000
62	2.60683299	2.48188000	11.01270833	1.98200000	13.49331000	0.51000000	0	2.62000000
63	0.81649658	1.85250000	2.33581962	2.03700000	21.74979000	0.29100000	1	2.25000000
64	0.81649658	2.02890000	1.61454047	2.15800000	25.14231000	0.41300000	1	2.01000000
65	0.81649658	2.42789000	2.39231824	2.64200000	20.51580000	0.56500000	0	2.47000000
66	1.33333333	2.59380000	1.35013717	2.57600000	29.27866000	0.53200000	1	2.19000000
67	8.07522665	4.18957000	12.41230678	5.51900000	41.55309000	2.09700000	1	2.23000000
68	7.46243974	3.20361000	12.39435185	2.73400000	38.02496000	2.07300000	0	4.07000000
69	5.30142366	5.28269000	10.96594300	3.16300000	44.89904000	1.61200000	1	5.03000000
70	4.84090128	2.93840000	2.24884259	6.24400000	39.34622000	1.61600000	0	4.15000000
71	6.45110142	2.43039000	12.10129630	4.07400000	33.88006000	1.79000000	0	3.34000000
72	4.82469569	2.00839000	11.54421249	3.18000000	24.16500000	0.75200000	1	2.65000000
73	5.73537930	3.28059000	11.98253039	4.49000000	32.62882000	0.72200000	1	4.46000000
74	5.86270050	3.56879000	11.94474482	5.03400000	32.62882000	0.75200000	1	4.61000000
75	6.86270050	5.12919000	12.18779156	7.31000000	43.30742000	0.75200000	1	6.57000000
76	5.39393419	3.99300000	2.17824074	7.81400000	49.87435000	2.12800000	0	4.35000000
77	5.39393419	3.79319000	4.33133409	4.90400000	43.64672000	2.10600000	0	3.32000000
78	5.13947547	6.06150000	2.38548459	5.64700000	37.38780000	2.20400000	1	7.48000000
79	4.12473677	3.50779000	10.63415666	2.31300000	32.13672000	1.18100000	1	3.36000000
80	4.08023187	0.96559000	10.83287037	1.96300000	16.66491000	1.17100000	0	2.07000000
81	7.35468279	3.62939000	12.24324397	6.59200000	40.96148000	1.37600000	1	4.84000000
82	7.01496572	3.11199000	12.09656541	4.73700000	37.16241000	0.79200000	1	4.15000000
83	4.98206207	3.51909000	10.78185941	4.81100000	32.11085000	1.36400000	1	3.16000000
84	7.12082441	2.98809000	12.27532236	2.81700000	46.89406000	2.01000000	1	2.53000000
85	5.09118476	3.11860000	2.21759259	7.02900000	41.06258000	1.75200000	0	4.02000000

Table S3. *Cont.*

ID	*SP3*	*CrippenLogP*	*gmax*	*XLogP*	*DCW*	*MLFER.E*	*SubFP302*	$\log K_{ow}Epi$
86	5.09118476	3.24439000	3.37796296	4.07800000	40.73897000	1.96200000	0	3.23000000
87	4.49735402	2.10149000	10.83461782	4.22800000	32.83889000	1.72800000	1	1.72000000
88	4.49735402	2.70079000	10.61239560	4.95600000	28.85481000	1.51200000	1	2.60000000
89	3.11406879	3.64680000	2.28097394	3.08400000	35.86276000	1.06100000	0	3.93000000
90	4.97606774	5.02979000	9.84471879	2.31300000	39.64281000	1.30400000	0	4.74000000
91	3.34344480	3.72299000	9.43070645	2.12100000	28.69064000	1.03400000	0	3.45000000
92	3.03412991	1.72129000	10.06731481	2.57800000	15.59586000	1.11000000	1	1.91000000
93	5.67448844	3.37258000	10.68268849	4.22300000	27.17985000	1.38400000	1	3.67000000
94	3.58746766	1.05139000	10.46287037	1.69200000	14.95817000	0.89600000	1	1.07000000
95	2.81349615	1.77139000	8.98550926	1.38900000	12.64134000	0.78200000	1	1.34000000
96	3.93315398	2.86782000	2.15740741	6.26100000	40.79846000	1.40000000	0	3.72000000
97	3.93315398	2.91599000	9.37120370	3.70900000	27.48231000	1.56500000	0	2.69000000
98	5.60816713	2.05039000	11.98726379	2.35100000	38.95530000	1.97700000	1	1.31000000
99	4.78679992	2.41050000	9.99185185	5.35400000	35.05573000	1.43800000	1	2.71000000
100	3.11525137	2.20459000	5.01120370	1.90800000	14.94304000	0.70000000	1	1.64000000
101	3.46632650	2.83980000	2.12037037	5.93800000	34.65443000	1.39200000	0	3.17000000
102	3.46632650	2.63999000	4.18120370	3.02800000	28.42679000	1.37000000	0	2.14000000
103	3.80187170	2.86782000	2.20370370	6.26100000	40.79846000	1.40000000	0	3.72000000
104	2.59263896	1.75260000	2.08333333	2.60600000	22.59221000	0.90600000	1	2.17000000
105	5.54213196	2.28929000	4.48251743	2.95000000	33.96500000	1.78900000	1	2.55000000
106	5.94414318	4.98149000	5.84977499	2.89200000	49.94839000	2.34800000	0	3.21000000
107	7.22165527	5.14620000	2.27490741	9.69000000	65.09428000	2.86400000	0	5.52000000
108	3.96632650	3.40200000	2.12037037	7.29200000	39.63152000	1.52800000	0	3.76000000
109	5.34399088	3.59338000	4.51518519	3.60800000	34.72924000	2.08400000	0	2.29000000
110	4.78769370	3.67469000	5.60886516	3.12200000	38.99622000	2.07800000	0	1.92000000
111	5.07580220	3.57299000	11.09317996	6.16100000	39.51206000	1.67500000	1	3.44000000
112	4.52305277	3.01079000	11.08905848	4.80700000	34.53497000	1.53900000	1	2.85000000
113	3.09851832	1.44159000	10.79120370	2.98700000	17.61042000	0.70400000	1	1.83000000

Table S3. *Cont.*

ID	*SP3*	*CrippenLogP*	*gmax*	*XLogP*	*DCW*	*MLFER.E*	*SubFP302*	$\log K_{ow}Epi$
114	5.00685252	4.23338000	10.67346163	2.54800000	31.61925000	1.20900000	1	3.68000000
115	4.30905462	3.84488000	10.34244312	2.02200000	30.43210000	1.22400000	1	3.26000000
116	3.19858470	1.83169000	11.02245370	3.41000000	19.16912000	0.70400000	1	2.32000000
117	4.88443100	3.37498000	11.59185563	5.53000000	28.54984000	1.32800000	1	3.04000000
118	3.60926831	1.85971000	11.13375378	3.73300000	20.52158000	0.71200000	1	2.87000000
119	4.09520854	2.57078000	5.22890779	2.93000000	15.22110000	1.04100000	1	3.45000000
120	3.72395631	2.62081000	10.23393814	1.94200000	23.53672000	0.96200000	1	2.52000000
121	3.72395631	3.19148000	10.24729163	1.92600000	24.95601000	1.08900000	1	2.62000000
122	3.21632650	1.28638000	3.88259259	1.95700000	30.16902000	1.39600000	0	1.17000000
123	3.21632650	2.90130000	2.41203704	3.36200000	29.19357000	1.37200000	0	2.99000000
124	2.54033641	1.74264000	2.12037037	4.70800000	27.66211000	0.67200000	0	3.09000000
125	2.54033641	1.84552000	8.91925926	2.15600000	14.34596000	0.83700000	0	2.06000000
126	2.54033641	2.36802000	2.19307270	3.21500000	26.99416000	0.79900000	0	3.18000000
127	2.54033641	2.99340000	2.23396776	2.98800000	30.38667000	0.92600000	0	3.28000000
128	2.54033641	2.41619000	8.96015432	1.92900000	17.73847000	0.86400000	0	2.16000000
129	2.86036622	1.77066000	2.20370370	5.03100000	29.74568000	0.68000000	0	3.63000000
130	2.86036622	3.68389000	5.57184842	1.95800000	28.94097000	1.20100000	0	2.37000000
131	2.86036622	3.06959000	8.98184842	2.02500000	23.21456000	1.09900000	0	2.80000000
132	3.42994914	1.79868000	2.24074074	5.35400000	31.82926000	0.68800000	0	4.18000000
133	3.42994914	4.30020000	2.46793553	3.18000000	41.33884000	1.19600000	0	4.57000000
134	3.42994914	3.72299000	9.30978052	2.12100000	28.69064000	1.13400000	0	3.45000000
135	1.39384685	2.38360000	1.70846193	3.05500000	30.35115000	0.77600000	1	2.68000000
136	3.16114088	1.94650000	11.91231481	2.88000000	25.90516000	0.63900000	1	2.04000000
137	2.59263896	1.39880000	9.02370370	2.86900000	20.48999000	0.81400000	1	1.49000000
138	2.59263896	1.85759000	10.64537037	2.93100000	19.31505000	0.80300000	1	1.67000000
139	2.59263896	2.01569000	10.00648148	2.89400000	17.51861000	0.93700000	1	1.81000000
140	2.91976578	1.59789000	10.10314815	1.65300000	18.32902000	1.21200000	1	1.47000000
141	4.08701484	2.90469000	10.41776084	1.84500000	29.28119000	1.48200000	1	2.76000000

Table S3. *Cont.*

ID	*SP3*	*CrippenLogP*	*gmax*	*XLogP*	*DCW*	*MLFER.E*	*SubFP302*	$\log K_{ow}Epi$
142	4.64492371	0.88759000	10.50537037	1.80700000	19.25062000	1.33800000	1	1.51000000
143	4.64492371	1.55009000	10.31092593	2.45700000	21.68371000	1.49900000	1	1.45000000
144	3.58154730	3.32249000	10.30153285	2.87500000	28.47078000	1.20700000	1	3.10000000
145	3.64094686	1.78289000	10.15870370	2.57000000	19.60116000	1.21800000	1	1.63000000
146	4.30806041	1.59889000	11.13375378	2.66400000	21.77148000	0.98500000	1	2.14000000
147	3.00332256	1.38121000	10.31222600	2.98900000	17.39229000	0.78400000	1	2.42000000
148	3.00332256	1.05879000	10.23059335	1.07000000	11.91184000	0.94900000	1	1.39000000
149	3.00332256	1.59789000	10.07696523	1.65300000	18.32902000	1.21200000	1	1.47000000
150	3.00332256	1.72129000	10.03614890	1.72000000	15.59586000	1.11000000	1	1.91000000
151	2.30209514	2.24900000	2.16203704	4.95400000	23.81978000	0.66400000	1	3.03000000
152	2.30209514	2.14689000	3.63092593	4.95400000	19.61575000	0.79100000	1	2.89000000
153	2.30209514	0.83940000	8.53759259	2.40900000	18.40642000	0.82900000	1	1.08000000
154	2.30209514	1.01949000	8.24759259	0.74800000	13.64400000	0.78600000	0	0.35000000
155	2.30209514	1.72830000	3.02694444	2.36700000	20.29050000	0.85600000	1	1.62000000
156	2.30209514	2.19599000	4.91416667	2.56300000	14.56409000	0.67800000	1	2.07000000
157	5.20134390	4.58068000	4.35412558	3.31800000	35.93798000	2.38100000	1	3.64000000
158	3.65447383	3.49498000	11.21419438	3.19200000	28.06742000	1.01600000	1	3.30000000
159	3.56793442	1.97818000	11.12704082	2.03500000	20.34704000	1.00100000	1	1.38000000
160	6.33510783	3.22180000	2.20356198	4.94900000	44.36836000	1.78800000	1	4.37000000
161	4.93752361	3.36249000	5.62339065	3.69100000	35.16153000	1.83800000	1	2.18000000
162	4.11615641	3.97969000	5.57833333	5.10600000	27.79189000	1.30200000	1	4.05000000
163	3.24192155	2.02609000	10.99023526	3.82400000	22.50846000	0.70400000	1	2.57000000
164	3.24192155	2.45308000	10.87912415	2.84700000	19.20397000	0.89600000	1	2.24000000
165	3.73205081	3.37380000	2.31861111	6.73800000	32.59034000	0.68000000	1	5.11000000
166	6.86582751	6.29989000	11.79776422	7.35600000	48.86713000	0.09000000	1	8.12000000
167	4.43265299	3.89738000	4.10148148	6.10000000	39.83014000	1.40500000	1	4.11000000
168	2.42166830	2.63910000	2.20138889	5.52300000	26.41611000	0.66400000	1	3.52000000
169	2.42166830	0.61640000	8.47968821	1.12300000	12.27770000	0.80700000	1	0.38000000

Table S3. *Cont.*

ID	*SP3*	*CrippenLogP*	*gmax*	*XLogP*	*DCW*	*MLFER.E*	*SubFP302*	$\log K_{ow}Epi$
170	2.52948273	1.54759000	10.16870748	3.08000000	17.90505000	0.77600000	1	1.43000000
171	2.52948273	2.47898000	10.50204082	2.25300000	17.06150000	0.99500000	1	1.10000000
172	2.94016633	3.24148000	10.60158081	2.47600000	21.88362000	1.30300000	1	1.99000000
173	2.69067070	3.02920000	2.22513889	6.09200000	32.56014000	0.66400000	1	4.01000000
174	2.33195123	2.75379000	10.78398101	3.19300000	17.14955000	0.08000000	1	3.32000000
175	2.58195123	3.14389000	10.85352190	3.76200000	19.45126000	0.08000000	1	3.81000000
176	2.30453045	3.13959000	5.45264403	2.20000000	20.70240000	1.23900000	0	1.97000000
177	2.30453045	2.52529000	8.78264403	2.26700000	14.97599000	1.13700000	0	2.40000000
178	2.30453045	1.74264000	2.12037037	4.70800000	23.60165000	0.67200000	0	3.09000000
179	2.30453045	1.79081000	8.75666667	2.36700000	14.34596000	0.83700000	0	2.06000000
180	2.30453045	2.99340000	2.23396776	2.98800000	30.38667000	0.92600000	0	3.28000000
181	2.30453045	3.03049000	5.44484225	1.86200000	23.46488000	1.06600000	0	1.72000000
182	2.30453045	2.41619000	8.77484225	1.92900000	17.73847000	0.96400000	0	2.16000000
183	2.30453045	2.40511000	5.42666667	2.30000000	20.07237000	0.93900000	0	1.62000000
184	0.98559856	0.62400000	4.89853395	0.61200000	11.92476000	0.37300000	1	0.63000000
185	0.50000000	1.77620000	1.65715021	2.41600000	19.02717000	0.65200000	1	2.01000000
186	0.50000000	0.53138000	9.05555556	0.29300000	1.66962000	0.29800000	1	0.19000000
187	0.50000000	1.46400000	1.55572702	1.74000000	19.66622000	0.30600000	1	1.83000000
188	2.19858470	1.74264000	2.16666667	4.70800000	23.60165000	0.67200000	0	3.09000000
189	2.19858470	1.79081000	8.81305556	2.36700000	14.34596000	0.83700000	0	2.06000000
190	2.19858470	2.41619000	8.83922840	1.92900000	17.73847000	0.96400000	0	2.16000000
191	2.19858470	2.40511000	5.45861111	2.30000000	20.07237000	0.93900000	0	1.62000000
192	2.19858470	1.46839000	8.65305556	0.87000000	10.33963000	1.00200000	0	1.03000000
193	1.06066017	2.95280000	5.77040466	2.58800000	21.48280000	0.26600000	1	3.73000000
194	2.41421356	1.77066000	2.18750000	5.03100000	25.68522000	0.68000000	0	3.63000000
195	2.41421356	1.81883000	8.99388889	2.69000000	16.42953000	0.84500000	0	2.61000000
196	2.41421356	3.64680000	2.35907750	3.08400000	35.86276000	1.06100000	0	3.93000000
197	1.89384685	2.44910000	2.18724280	3.44100000	22.14811000	0.96400000	0	2.88000000

Table S3. *Cont.*

ID	*SP3*	*CrippenLogP*	*gmax*	*XLogP*	*DCW*	*MLFER.E*	*SubFP302*	$\log K_{ow}Epi$
198	1.89384685	1.71462000	2.08333333	4.38500000	21.51808000	0.66400000	0	2.54000000
199	1.89384685	2.34000000	2.15603567	3.10300000	24.91059000	0.79100000	0	2.64000000
200	1.89384685	1.76279000	8.63222222	2.04400000	12.26238000	0.82900000	0	1.51000000
201	0.70710678	0.74520000	5.13902778	0.78400000	13.21149000	0.22900000	1	0.83000000
202	1.20710678	1.55900000	8.29288265	1.98900000	12.08849000	0.20500000	1	1.82000000
203	0.94280904	2.17260000	5.39540466	1.75400000	17.09751000	0.27800000	1	1.41000000
204	1.45710678	1.94910000	8.36558673	2.55800000	14.39020000	0.20500000	1	2.31000000
205	1.70710678	2.33920000	8.42278195	3.12700000	16.69190000	0.20500000	1	2.81000000
206	2.20710678	3.11940000	8.50701264	4.26500000	21.29532000	0.20500000	1	3.79000000
207	2.70710678	3.89960000	8.56606351	5.40300000	25.89873000	0.20500000	1	4.77000000
208	4.24269231	2.29078000	11.03032596	2.65200000	20.97503000	0.84900000	1	1.90000000
209	3.06327271	2.02868000	10.45021640	1.26000000	20.57295000	1.20900000	1	1.35000000
210	8.54344624	3.39358000	11.82003337	1.28500000	37.75930000	1.53200000	1	3.14000000
211	9.30235191	3.71469000	11.79924855	0.83900000	46.99474000	1.91400000	0	3.50000000
212	7.34388543	5.02570000	11.62163895	6.11000000	59.56945000	2.01100000	1	5.81000000
213	5.32280178	3.06969000	11.48868490	2.77700000	32.08491000	1.64100000	1	2.35000000
214	3.20450702	1.21748000	10.55628071	1.68400000	16.18597000	0.71200000	1	1.36000000
215	4.52275210	4.62929000	10.84128944	3.06700000	39.42295000	1.47700000	1	4.39000000
216	8.48372753	6.40139000	12.56978223	9.04200000	56.78793000	0.72200000	1	8.39000000
217	7.86270050	6.68959000	12.35181352	9.58600000	56.04837000	0.75200000	1	8.54000000
218	4.97606774	5.60700000	1.87120713	3.37200000	52.29102000	1.46600000	0	5.86000000
219	4.97606774	2.60287000	11.55765432	2.08000000	20.01264000	1.33800000	0	2.22000000
220	5.15594081	2.19471000	10.53175926	2.56900000	23.76729000	1.50700000	1	1.99000000
221	4.78679992	2.88599000	11.84629630	5.67700000	36.08357000	1.42700000	1	3.15000000
222	7.82715413	5.30878000	4.68977466	5.99400000	54.92626000	2.95600000	0	4.06000000
223	5.34399088	3.99300000	2.24074074	7.81400000	49.87435000	2.12800000	0	4.35000000
224	4.42175420	3.57998000	10.57831015	2.45200000	27.25772000	1.07400000	1	3.03000000
225	3.60926831	1.53729000	11.05212113	1.81400000	17.98938000	0.87700000	1	2.49000000

Table S3. *Cont.*

ID	*SP3*	*CrippenLogP*	*gmax*	*XLogP*	*DCW*	*MLFER.E*	*SubFP302*	$\log K_{ow}Epi$
226	2.54033641	1.46839000	8.66925926	1.29900000	10.33963000	0.93300000	0	1.03000000
227	2.86036622	3.64680000	2.39000343	3.08400000	35.86276000	1.06100000	0	3.93000000
228	2.86036622	3.06959000	9.10277435	2.02500000	23.21456000	0.99900000	0	2.80000000
229	2.59263896	1.75260000	2.08333333	2.60600000	44.36836000	0.90600000	1	2.17000000
230	5.49744232	2.02629000	11.96176120	2.00700000	31.39062000	1.01800000	1	2.29000000
231	4.64492371	0.28829000	10.72759259	1.07900000	23.45790000	1.55400000	1	0.37000000
232	5.68915432	2.66512000	10.66934274	3.59200000	25.64718000	1.55000000	1	3.30000000
233	3.57091705	2.24618000	10.61334089	1.92900000	19.14405000	1.27600000	1	1.64000000
234	3.53191804	2.19898000	4.20519383	1.07500000	21.28174000	1.86500000	1	2.40000000
235	4.11615641	4.03029000	3.30388889	4.91000000	31.71009000	1.67200000	1	3.29000000
236	3.33422058	2.84158000	11.08954082	3.30700000	23.10432000	0.88100000	1	2.66000000
237	4.43265299	3.72569000	3.11092593	5.67000000	34.25511000	1.67200000	1	3.06000000
238	2.30453045	−1.13632000	10.19597222	0.19000000	15.79240000	0.78400000	0	−0.89000000
239	2.08195123	2.36369000	10.69565193	2.62400000	13.16637000	0.08000000	1	2.83000000
240	1.50000000	0.03320000	4.94444444	−0.38000000	6.82763000	0.27400000	0	−0.32000000
241	0.00000000	2.29860000	1.46450617	2.75000000	18.87350000	0.70100000	0	1.70000000
242	1.33333333	2.99439000	1.89849108	3.02600000	28.80875000	0.70000000	0	2.97000000
243	11.63965014	5.27308000	13.06556894	7.50400000	74.56275000	3.86200000	0	6.28000000
244	6.73792507	4.58400000	2.21240741	8.33600000	60.11719000	2.72800000	0	4.93000000
245	4.62456292	1.22819000	11.22463341	2.33400000	19.56159000	0.75200000	1	1.66000000
246	5.09118476	3.51209000	5.65240741	4.27400000	39.31121000	1.86700000	0	3.71000000
247	5.09118476	4.05450000	2.31224280	4.60500000	43.06858000	2.10800000	0	4.17000000
248	5.42431786	3.07358000	12.20684156	2.26800000	34.17278000	1.49700000	1	2.74000000
249	5.42431786	3.22689000	12.09772119	3.80200000	40.34508000	1.81500000	1	2.84000000
250	3.87790778	2.24219000	10.71499657	1.19500000	26.84809000	1.32100000	1	1.90000000
251	3.93315398	3.53029000	5.75620370	3.64200000	33.20872000	1.66700000	0	2.25000000
252	3.35349264	2.06080000	2.42909979	1.54800000	37.87716000	2.02000000	1	1.70000000
253	3.74754690	2.97598000	4.29561728	1.99500000	25.88516000	1.83500000	1	3.24000000

Table S3. *Cont.*

ID	*SP3*	*CrippenLogP*	*gmax*	*XLogP*	*DCW*	*MLFER.E*	*SubFP302*	$\log K_{ow}Epi$
254	4.93752361	3.54358000	5.64705320	2.95500000	37.66243000	2.16800000	1	2.46000000
255	4.46059291	0.57598000	11.60350687	−0.34600000	4.12392000	0.53100000	1	−0.10000000
256	1.38502781	1.88519000	9.87412415	1.88000000	8.56295000	0.15200000	1	2.05000000
257	1.95710678	2.72930000	8.46895479	3.69600000	18.99361000	0.20500000	1	3.30000000
258	14.38675135	4.85209000	13.82473237	3.72600000	68.27248000	2.45100000	0	3.86000000
259	4.58645442	0.18309000	10.76296296	−0.79000000	11.79915000	0.63400000	1	1.89000000
260	5.34399088	3.06328000	4.49120370	2.38900000	36.57381000	2.03400000	0	2.00000000
261	3.47673330	2.48910000	3.48682099	2.12700000	30.90489000	1.94600000	0	1.83000000
262	3.00332256	0.93539000	10.27140967	1.00300000	17.37005000	1.05100000	1	0.96000000
263	2.62234839	1.77139000	8.86416667	1.38900000	12.64134000	0.85100000	1	1.59000000
264	3.97861802	2.63158000	11.22219230	1.92000000	25.17637000	1.13600000	1	2.03000000
265	2.71277874	1.77139000	8.79663265	1.38900000	12.64134000	0.85100000	1	1.59000000
266	0.00000000	−0.41040000	3.00000000	−0.38600000	9.13272000	0.38600000	0	−0.28000000
267	0.50000000	1.86139000	2.23628258	2.25800000	15.03971000	0.43000000	0	1.98000000
268	10.51009060	6.89040000	2.32513605	12.08800000	90.55705000	4.20000000	0	7.28000000
269	9.99886610	6.32820000	2.26916667	10.73400000	85.57996000	4.06400000	0	6.70000000
270	8.62962008	5.73720000	2.24657407	10.21200000	75.33712000	3.46400000	0	6.11000000
271	9.98918639	6.32820000	2.37444444	10.73400000	85.57996000	4.06400000	0	6.70000000
272	8.87962008	5.55079000	3.56226002	7.83000000	77.79186000	3.43400000	0	5.58000000
273	8.61558946	5.73720000	2.24657407	10.21200000	75.33712000	3.46400000	0	6.11000000
274	8.62555263	5.73720000	2.34027778	10.21200000	75.33712000	3.46400000	0	6.11000000
275	6.73259630	4.58400000	2.21990741	8.33600000	60.11719000	2.72800000	0	4.93000000
276	8.62400783	5.73720000	2.33277778	10.21200000	75.33712000	3.46400000	0	6.11000000
277	4.84090128	3.03199000	2.17824074	6.46000000	35.57003000	1.75200000	0	3.94000000
278	7.32715413	5.14620000	2.23611111	9.69000000	65.09428000	2.86400000	0	5.52000000
279	9.21295342	6.29940000	2.35648148	11.56600000	80.31421000	3.60000000	0	6.70000000
280	7.27847215	4.94639000	4.72781557	6.53200000	57.52172000	2.84200000	0	4.49000000
281	7.03127828	4.27180000	2.26736111	8.90500000	56.28251000	2.48800000	0	5.19000000

Table S3. *Cont.*

ID	*SP3*	*CrippenLogP*	*gmax*	*XLogP*	*DCW*	*MLFER.E*	*SubFP302*	$\log K_{ow}Epi$
282	8.97137179	5.55079000	3.66398148	7.40800000	71.17882000	3.43400000	0	5.58000000
283	5.34399088	3.79319000	4.57768519	4.65600000	42.30180000	2.10600000	0	3.32000000
284	5.32280178	2.58239000	10.44850940	3.48000000	23.56361000	1.54200000	1	2.75000000
285	3.09099026	3.39830000	6.03570484	3.80900000	35.70788000	1.28300000	1	3.62000000
286	3.34099026	3.44080000	6.05893490	4.16700000	38.00959000	1.28300000	1	4.07000000
287	3.98449012	2.67120000	11.76430384	2.26600000	20.96995000	0.93500000	1	1.60000000
288	3.88128372	1.77480000	11.71716859	−0.65900000	22.37012000	0.92000000	1	−1.03000000
289	9.63646194	5.12258000	2.65373329	4.15600000	54.65385000	1.83000000	0	6.75000000
290	5.03568748	1.48728000	11.81215278	1.49700000	24.62283000	1.29000000	1	1.68000000
291	4.50447159	1.09718000	11.65326389	1.13900000	22.32112000	1.29000000	1	1.19000000
292	4.97606774	3.64440000	1.35354595	3.83400000	48.31834000	0.99200000	0	4.26000000
293	4.97606774	3.64440000	1.35354595	3.83400000	48.31834000	0.99200000	0	4.26000000
294	3.88818767	2.11728000	12.65378307	1.78300000	20.29106000	0.88300000	1	1.58000000
295	4.54996918	3.28498000	11.34684587	2.01600000	30.65245000	1.27100000	1	2.67000000
296	5.02725307	3.21658000	11.45309587	1.92500000	33.74212000	1.06500000	1	2.91000000
297	2.94016633	1.51438000	12.28015448	1.12300000	16.37911000	0.95600000	1	0.91000000
298	5.50829362	3.84001000	6.07164609	4.29600000	32.62336000	1.21800000	1	3.86000000
299	4.52569461	0.66078000	11.42615851	−0.01200000	2.40404000	0.31600000	1	0.76000000
300	2.84973598	2.61808000	12.46628307	2.00100000	19.76080000	0.87700000	1	1.30000000
301	3.81229215	3.15668000	12.19260818	3.15600000	28.98600000	0.70300000	1	2.06000000
302	2.94016633	2.61808000	12.31140448	2.00100000	19.76080000	0.87700000	1	1.30000000
303	3.96632650	2.59719000	4.18648148	2.73800000	27.17624000	1.48400000	0	1.38000000
304	7.00575134	3.23219000	8.29920824	4.01500000	46.14536000	2.08500000	1	2.98000000
305	3.21632650	2.17530000	2.23842593	4.93700000	30.27033000	0.88000000	0	3.47000000
306	7.57453897	3.45249000	12.38396794	4.15900000	44.36071000	1.77100000	1	3.99000000
307	3.11406879	1.77066000	2.15740741	5.03100000	25.68522000	0.68000000	0	3.63000000
308	4.97606774	5.64409000	6.10471879	2.24600000	45.36922000	1.60600000	0	4.30000000
309	4.65106752	0.88759000	10.46444822	1.80700000	20.72474000	1.33800000	1	1.51000000

Table S3. *Cont.*

ID	*SP3*	*CrippenLogP*	*gmax*	*XLogP*	*DCW*	*MLFER.E*	*SubFP302*	$\log K_{ow}Epi$
310	3.06327271	3.13238000	10.67243862	2.13800000	24.64610000	1.13000000	1	1.18000000
311	2.84973598	2.50700000	10.61334089	2.57600000	21.04469000	1.00300000	1	1.65000000
312	1.83195123	1.97359000	10.57972222	2.05500000	10.86466000	0.08000000	1	2.34000000
313	2.19858470	2.99340000	2.31207133	2.98800000	30.38667000	0.92600000	0	3.28000000
314	0.70710678	1.90389000	2.27040466	2.09900000	17.76390000	0.43000000	1	2.29000000
315	2.86036622	3.68389000	5.64777435	1.95800000	28.94097000	1.20100000	0	2.37000000
316	2.91976578	1.72129000	10.04759259	1.72000000	15.59586000	1.11000000	1	1.91000000
317	5.39632185	4.45528000	11.85291446	3.36600000	38.81122000	1.27100000	1	4.15000000
318	0.50000000	−0.20750000	3.56430041	1.61800000	12.55296000	0.62500000	0	1.30000000
319	4.97487373	4.34650000	6.18261072	4.97800000	44.93347000	1.86600000	1	5.00000000
320	3.11406879	3.06959000	9.16716049	2.02500000	23.21456000	0.99900000	0	2.80000000
321	4.38697001	2.89584000	2.24074074	6.58400000	42.88204000	1.40800000	0	4.26000000
322	3.00332256	2.77819000	10.13686697	3.11700000	20.23221000	1.24500000	1	2.70000000
323	2.84973598	2.02868000	10.40447216	1.26000000	21.26442000	1.20900000	1	1.35000000
324	3.06327271	3.13238000	10.67243862	2.13800000	24.64610000	1.13000000	1	1.18000000
325	2.84973598	3.13238000	10.62669438	2.13800000	24.64610000	1.13000000	1	1.74000000
326	2.41421356	3.06959000	9.04623457	2.02500000	23.21456000	1.09900000	0	2.80000000
327	1.89384685	2.29120000	2.19167901	3.64100000	19.27435000	1.30100000	0	3.16000000
328	0.00000000	2.86510000	1.43201303	3.54000000	24.34959000	0.77100000	0	2.62000000
329	1.73205081	1.07579000	10.36574074	0.30200000	19.46013000	0.67700000	1	0.83000000
330	0.81649658	−1.11751000	9.47916667	−1.16800000	4.36875000	0.39600000	1	−1.09000000
331	3.11406879	3.68389000	5.76364198	1.95800000	28.94097000	1.20100000	0	2.37000000
332	4.19739619	4.95360000	2.51494170	3.27600000	46.81493000	1.33100000	0	5.22000000
333	3.34344480	3.72299000	9.18885460	2.12100000	28.69064000	1.23400000	0	3.45000000
334	3.03412991	1.40729000	10.67015432	1.61200000	19.97225000	1.12700000	1	0.65000000
335	3.58746766	0.52109000	10.64398148	1.40300000	21.37535000	1.27300000	1	−0.18000000
336	3.44496681	1.35679000	11.27037037	2.59800000	19.09958000	0.91900000	1	0.73000000
337	5.67953608	4.68349000	5.76794155	5.72900000	48.42865000	2.40300000	0	3.43000000

Table S3. *Cont.*

ID	*SP3*	*CrippenLogP*	*gmax*	*XLogP*	*DCW*	*MLFER.E*	*SubFP302*	$\log K_{ow}Epi$
338	3.09851832	1.01459000	10.90231481	2.45600000	21.59451000	0.94100000	1	1.20000000
339	5.25086945	1.36609000	11.32689815	2.55100000	25.32815000	1.26600000	1	1.95000000
340	5.25086945	1.36609000	11.32689815	2.55100000	25.32815000	1.26600000	1	1.95000000
341	3.00332256	0.78191000	10.53444822	2.26100000	21.37637000	1.00000000	1	1.29000000
342	3.70211466	0.52109000	10.53444822	1.19200000	21.37535000	1.27300000	1	0.56000000
343	2.84973598	3.24148000	10.63242630	2.47600000	21.88362000	1.30300000	1	1.99000000
344	2.71277874	2.27702000	2.17592593	5.27700000	29.74568000	0.67200000	1	3.58000000
345	2.41421356	3.68389000	5.61179012	1.95800000	28.94097000	1.20100000	0	2.37000000
346	1.06066017	2.59540000	1.65252058	2.81100000	30.61839000	0.51200000	1	2.93000000
347	5.68632384	3.66499000	11.17532572	1.65400000	35.38839000	1.43600000	1	3.65000000
348	3.70183179	4.30020000	2.35890604	3.18000000	41.33884000	1.19600000	0	4.57000000
349	3.70183179	4.33729000	5.77478052	2.05400000	34.41705000	1.33600000	0	3.01000000
350	4.19739619	4.99069000	5.90178669	2.15000000	39.89314000	1.47100000	0	3.65000000
351	3.34344480	4.30020000	2.43700960	3.18000000	41.33884000	1.19600000	0	4.57000000
352	3.64094686	0.52109000	10.57537037	1.19200000	16.57872000	1.27300000	1	0.56000000
353	5.45028432	2.78249000	12.91548611	1.90500000	18.08688000	0.66100000	1	3.34000000
354	3.06327271	1.51438000	12.67967593	1.12300000	15.68765000	0.95600000	1	0.91000000
355	2.94016633	1.51438000	12.28015448	1.12300000	15.68765000	0.95600000	1	0.91000000
356	3.97119712	1.55418000	4.94594671	0.61000000	14.44228000	1.75200000	1	2.73000000
357	1.99156383	1.10859000	10.53194444	0.69800000	10.33909000	0.29500000	1	0.50000000
358	3.34344480	1.90156000	9.28092593	2.80200000	18.51311000	0.85300000	0	3.15000000
359	4.13940532	4.17588000	11.08990142	2.48700000	32.42390000	1.26500000	1	2.88000000
360	6.29981731	3.65959000	11.42889645	6.41800000	44.27445000	2.24800000	1	4.23000000
361	7.36457423	2.12959000	12.19865569	2.93300000	31.62029000	2.30900000	1	2.48000000
362	6.36388292	3.95709000	12.34056170	4.96400000	39.76116000	1.81900000	1	4.12000000
363	2.86768668	2.41459000	11.54367798	2.28800000	21.48317000	0.62500000	1	3.02000000
364	2.84973598	1.51438000	12.42546674	1.12300000	15.68765000	0.95600000	1	0.91000000
365	5.89193390	4.02102000	2.26157407	8.13700000	51.95793000	2.13600000	0	4.89000000

Table S3. *Cont.*

ID	*SP3*	*CrippenLogP*	*gmax*	*XLogP*	*DCW*	*MLFER.E*	*SubFP302*	$\log K_{ow}Epi$
366	6.29981731	4.16399000	11.87334089	6.68300000	47.75825000	2.27500000	1	4.03000000
367	5.23523778	4.52399000	6.15273941	5.39900000	51.87034000	2.04200000	1	5.44000000
368	3.96632650	3.73430000	2.27379630	6.82900000	33.55125000	0.85700000	1	4.81000000
369	4.07901155	3.06117000	4.39561392	1.80600000	27.24051000	2.05300000	1	3.32000000
370	6.87450882	3.04757000	11.92067492	6.06400000	34.73261000	2.16400000	1	3.31000000
371	4.73185917	4.62929000	10.71893683	3.06700000	39.42295000	1.47700000	1	4.39000000
372	4.17532126	3.45127000	4.44082226	2.22300000	29.54221000	2.04700000	1	3.77000000
373	4.92089426	3.33888000	11.65845238	3.72100000	29.43175000	1.21600000	1	3.44000000
374	3.34099026	1.89360000	11.71116722	1.37000000	25.03066000	0.87800000	1	1.01000000
375	3.34344480	3.72299000	9.37416667	2.12100000	28.69064000	1.13400000	0	3.45000000
376	4.22563538	3.40220000	2.25925926	6.83000000	39.03971000	1.40000000	1	4.21000000
377	4.21332607	3.86668000	6.18996410	5.31200000	39.77617000	1.56300000	1	4.02000000
378	5.54907779	1.78428000	11.65599108	1.77400000	30.94621000	1.79800000	1	1.58000000
379	5.23523778	3.74379000	5.76982274	4.55300000	47.26693000	2.04200000	1	4.46000000
380	6.04701118	3.12389000	12.36982237	6.21200000	35.25220000	1.52800000	1	2.86000000
381	6.68179791	4.43879000	12.22226993	3.46000000	34.59110000	1.43200000	1	3.81000000
382	3.15737056	1.63598000	10.75898526	1.89300000	17.39858000	1.02300000	1	1.17000000
383	9.46549057	4.37889000	6.14926838	3.03200000	46.98538000	1.81900000	0	4.56000000
384	3.27060293	2.80469000	11.65960769	2.85700000	23.78488000	0.62500000	1	3.51000000
385	4.24757747	3.40220000	2.19675926	6.83000000	39.03971000	1.40000000	1	4.21000000
386	3.24192155	3.25918000	11.10134637	3.07500000	23.77343000	0.99500000	1	2.08000000
387	4.20165201	2.94719000	11.89411733	2.97700000	26.61123000	0.81800000	1	3.81000000
388	3.25213997	2.93129000	8.70160494	2.33600000	29.47890000	1.07800000	0	2.83000000
389	4.97606774	4.08199000	9.55382373	1.68300000	32.24397000	1.27300000	0	3.61000000
390	6.11253258	3.14580000	9.53363568	6.16100000	45.52647000	2.30100000	1	3.43000000
391	3.55187170	2.25149000	9.09638889	2.91900000	19.25588000	1.05300000	0	2.99000000
392	3.76092395	3.23328000	11.15394274	3.77400000	24.89304000	0.89600000	1	3.22000000
393	5.36593209	2.21708000	11.15353080	2.48300000	22.25600000	1.05800000	1	2.30000000

Table S3. *Cont.*

ID	*SP3*	*CrippenLogP*	*gmax*	*XLogP*	*DCW*	*MLFER.E*	*SubFP302*	$\log K_{ow}Epi$
394	6.90275936	4.08699000	12.84483009	4.84500000	49.46840000	1.17600000	1	5.31000000
395	4.07901155	1.94268000	4.98118859	1.07000000	16.74399000	1.73700000	1	3.15000000
396	4.18682598	2.33118000	5.01643046	1.53000000	19.04569000	1.72200000	1	3.57000000
397	0.95710678	0.12040000	2.94791667	1.43000000	7.38642000	0.04000000	1	0.45000000
398	3.77790994	1.09868000	11.27202641	−0.34500000	11.27895000	0.75400000	1	−0.78000000
399	4.22595363	0.97988000	11.29903258	0.46300000	9.38613000	0.76500000	1	−0.67000000
400	3.78272388	2.85509000	9.43375000	2.04900000	20.28885000	1.39700000	0	3.39000000
401	5.92440181	5.41539000	11.86878391	5.88700000	37.02264000	1.47200000	1	5.86000000
402	5.61292987	2.11527000	11.85136180	2.82600000	32.03669000	1.62500000	1	0.76000000
403	4.45590190	2.56318000	11.32844230	1.82900000	23.99427000	0.93000000	1	2.26000000
404	4.21332607	0.77129000	11.79910872	1.46100000	11.93340000	0.66800000	1	0.32000000
405	6.07945191	5.61558000	10.53796112	4.76300000	42.07754000	1.85300000	1	4.32000000
406	6.44983090	6.26898000	10.59861956	4.85900000	47.55362000	1.98800000	1	4.96000000
407	6.68921137	3.84179000	11.86053035	2.29600000	39.99181000	1.29200000	1	4.24000000
408	7.03667321	4.08699000	12.83324403	5.05600000	49.46840000	1.17600000	1	5.31000000
409	5.30288319	4.10977000	9.05902592	1.87600000	41.77940000	1.50000000	0	3.66000000
410	3.63973247	2.58748000	4.25561050	1.53500000	23.10248000	1.85000000	1	2.82000000
411	4.59706269	3.00188000	4.43450617	2.19700000	27.92402000	1.90000000	1	3.86000000
412	4.48924826	2.61338000	4.39449938	1.73700000	25.62232000	1.91500000	1	3.44000000
413	4.20091038	2.66859000	10.91805213	2.33500000	26.92923000	1.06800000	1	2.14000000
414	4.73185917	2.29059000	10.77715014	−1.49800000	27.44146000	1.43400000	1	1.36000000
415	3.40286366	2.62760000	5.86257202	2.06500000	37.06525000	1.66700000	1	2.96000000
416	4.26497758	3.42778000	11.72071366	3.51500000	30.21149000	1.15700000	1	2.42000000
417	4.13940532	3.36978000	10.86767920	2.40500000	28.94010000	1.16600000	1	3.04000000
418	3.13668908	2.80469000	11.70256687	2.64600000	23.78488000	0.62500000	1	3.51000000
419	3.16114088	3.68050000	4.63983196	3.79100000	34.52050000	1.08600000	1	3.35000000
420	3.15737056	2.06298000	10.64787415	2.42400000	17.98792000	0.89600000	1	1.75000000
421	4.28236166	3.18787000	11.27752771	3.10300000	22.76433000	1.16000000	1	3.02000000

Table S3. *Cont.*

ID	*SP3*	*CrippenLogP*	*gmax*	*XLogP*	*DCW*	*MLFER.E*	*SubFP302*	$\log K_{ow}Epi$
422	2.94016633	2.13778000	10.37935858	1.59800000	17.81047000	1.38200000	1	1.60000000
423	2.84973598	2.02868000	10.40447216	1.26000000	20.57295000	1.20900000	1	1.35000000
424	6.61161118	4.92467000	11.50751384	3.80800000	38.78313000	1.78200000	1	4.08000000
425	5.45468425	3.58488000	11.80400794	4.01800000	31.73346000	1.20100000	1	3.86000000
426	3.09706269	2.90659000	11.83478909	2.88200000	26.08658000	0.59500000	1	3.85000000
427	3.40286366	2.06069000	10.91716049	1.49700000	25.44834000	1.26200000	1	0.90000000
428	4.77607048	1.76932000	11.03825310	2.80400000	25.20712000	1.19600000	1	2.87000000
429	4.78769370	4.70880000	2.23093252	5.37400000	50.58369000	1.79800000	0	5.05000000
430	4.43315398	4.05540000	2.21390604	6.12200000	45.10760000	1.66300000	0	4.40000000
431	4.29345332	4.05540000	2.27640604	6.33300000	45.10760000	1.66300000	0	4.40000000
432	6.04161840	3.28249000	12.59281659	5.54100000	42.56328000	1.12600000	1	3.78000000
433	7.00109309	3.93668000	10.68483231	6.44200000	40.53048000	2.15200000	1	4.47000000
434	5.20979284	3.17929000	11.43392337	5.70000000	35.53180000	1.44000000	1	3.75000000
435	5.62677957	3.66499000	11.16946168	1.65400000	35.38839000	1.43600000	1	2.13000000
436	3.51151749	3.78578000	10.72184587	2.23400000	30.12219000	1.26500000	1	2.39000000
437	3.63973247	2.01027000	9.19119426	0.55100000	23.11666000	1.88800000	1	2.09000000
438	4.85074384	2.65588000	12.34075633	2.93800000	34.94687000	0.70900000	1	2.35000000
439	3.44858470	2.16859000	11.57145576	1.94800000	22.00781000	0.83300000	1	2.91000000
440	7.84900179	8.06700000	1.87552882	4.59600000	78.46312000	2.47200000	0	8.33000000
441	4.00839760	3.63668000	12.07141454	3.44000000	27.42572000	0.99300000	1	4.08000000
442	4.41208788	4.20318000	12.19606810	3.82400000	31.56207000	1.12800000	1	4.57000000
443	5.12731945	4.57670000	11.80083507	3.93400000	35.93651000	1.12300000	1	4.18000000
444	7.04712711	4.41638000	12.08507623	4.19200000	46.49066000	2.38300000	1	4.29000000
445	7.52967273	4.59828000	11.93626342	4.25300000	44.03796000	1.38700000	1	5.57000000
446	3.51151749	2.68208000	10.49962365	1.35600000	26.04904000	1.34400000	1	2.00000000
447	15.00000000	6.22300000	1.43521862	6.35800000	79.25511000	2.57600000	0	7.01000000
448	3.56805416	1.53160000	10.73280187	2.74700000	16.74899000	0.85000000	1	1.72000000
449	3.73096416	1.12418000	10.55493040	3.42800000	17.10857000	1.02700000	1	1.62000000
450	5.68994288	2.24699000	11.45508729	2.22800000	25.84892000	2.37900000	0	3.37000000

Table S3. *Cont.*

ID	*SP3*	*CrippenLogP*	*gmax*	*XLogP*	*DCW*	*MLFER.E*	*SubFP302*	$\log K_{ow}Epi$
451	5.13947547	4.38499000	9.87007373	1.77300000	34.54568000	1.22200000	1	3.92000000
452	3.46289610	2.33989000	5.41278807	2.68100000	23.26995000	0.93100000	1	3.60000000
453	7.73820262	5.83669000	6.21332861	7.39400000	63.64857000	3.13900000	0	4.60000000
454	4.49873959	3.73159000	9.66306756	1.67700000	29.06959000	1.08700000	1	3.27000000
455	4.00729941	3.51139000	5.23680052	2.10000000	25.89522000	0.97000000	1	3.44000000
456	4.52245259	1.64235000	11.02269652	3.18200000	20.91614000	0.86600000	1	2.56000000
457	5.65634164	4.79389000	6.19184328	4.21600000	42.88450000	1.64400000	1	5.11000000
458	6.90099367	3.68098000	12.43797311	2.77000000	35.06785000	1.67400000	1	3.42000000
459	4.19739619	2.18557000	11.47197531	2.05900000	31.17336000	1.08300000	0	1.08000000
460	2.84973598	2.13778000	10.41020408	1.59800000	17.81047000	1.38200000	1	1.60000000
461	4.45590190	2.67228000	11.33283081	2.16700000	21.23179000	1.10300000	1	2.51000000
462	5.03225796	4.25619000	6.07425422	4.80600000	38.97303000	1.59900000	1	4.51000000
463	5.38908730	4.49960000	6.57051440	5.62600000	39.65954000	1.22000000	1	5.94000000
464	4.69251159	0.42538000	11.60816893	0.29700000	13.77705000	1.25100000	1	0.05000000
465	9.57648205	4.90508000	6.05128888	6.60900000	53.30104000	2.82800000	1	6.17000000
466	7.21697577	4.38309000	12.01933370	4.08100000	45.50625000	1.28300000	1	5.08000000
467	4.19739619	4.99069000	6.01765432	2.15000000	39.89314000	1.47100000	0	3.65000000
468	4.13940532	2.94278000	10.97879031	1.87400000	28.35075000	1.29300000	1	2.46000000
469	4.78525839	2.55480000	8.89905266	5.63900000	35.28363000	1.70100000	1	2.84000000
470	4.26776695	2.93920000	6.32384774	4.19400000	30.45272000	1.22000000	1	3.98000000
471	4.74476541	3.01708000	11.11102560	4.18200000	23.65411000	0.83500000	1	2.86000000
472	5.07917170	2.65077000	11.69435357	1.23400000	24.38162000	1.92800000	1	2.08000000
473	3.47762381	2.71638000	10.77252771	2.30900000	23.46401000	1.03100000	1	2.40000000
474	4.30639913	3.83977000	4.49190172	2.62400000	31.84392000	2.03800000	1	4.22000000
475	2.91976578	1.40159000	10.30882373	1.50600000	14.42796000	0.88900000	1	1.33000000
476	6.90275936	3.89359000	11.80444051	2.37600000	36.10482000	1.85900000	1	2.92000000
477	4.24757747	1.99260000	9.03561413	4.28500000	30.30654000	1.56500000	1	2.25000000
478	5.33859021	3.38017000	11.98217971	3.18700000	27.32649000	1.69800000	1	1.49000000

Table S3. *Cont.*

ID	*SP3*	*CrippenLogP*	*gmax*	*XLogP*	*DCW*	*MLFER.E*	*SubFP302*	$\log K_{ow}Epi$
479	6.03891366	3.14257000	12.00243433	1.96600000	21.82388000	1.75100000	1	1.41000000
480	3.13352596	2.43159000	5.55282250	1.41800000	22.10150000	1.08800000	1	1.80000000
481	3.51092395	2.84318000	11.03801304	3.20500000	22.59134000	0.89600000	1	2.73000000
482	5.65634164	4.01369000	6.00934328	3.37000000	38.28109000	1.64400000	1	4.13000000
483	4.64998668	1.33118000	11.84091821	1.38000000	27.38531000	1.11100000	1	1.75000000
484	3.73604218	2.97758000	4.30081883	1.95200000	25.88516000	1.84400000	1	3.27000000
485	8.57303800	6.40139000	12.23586607	8.62000000	55.76219000	0.72200000	1	8.39000000
486	8.30857091	1.04748000	11.05180143	0.65900000	30.55247000	1.90200000	1	0.87000000
487	4.05002905	0.23378000	11.51517778	−0.48800000	1.82222000	0.55300000	1	−0.31000000
488	2.86036622	3.16761000	5.55347737	2.31200000	22.78597000	1.24700000	0	2.51000000
489	5.16386739	5.36220000	2.38696819	5.04800000	56.05977000	1.93300000	0	5.69000000
490	3.78467723	3.14098000	10.74991300	1.69400000	25.02506000	1.15200000	1	1.83000000
491	4.42175420	3.00931000	10.56495666	2.46800000	23.86521000	0.94700000	1	2.94000000
492	3.84253526	3.63439000	5.20042181	3.46200000	29.65009000	1.10800000	1	3.89000000
493	3.97861802	2.00620000	11.21196854	2.35800000	21.78386000	1.00900000	1	1.93000000
494	4.38686631	1.98678000	11.25025943	1.38000000	20.07924000	1.02300000	1	1.46000000
495	4.39645541	2.33059000	11.30527435	1.51600000	29.14980000	1.24900000	1	2.60000000
496	4.18682598	3.44967000	4.42065172	2.26600000	29.54221000	2.03800000	1	3.73000000
497	6.58182735	2.45108000	11.85012515	3.41100000	19.94352000	0.92500000	1	2.89000000
498	4.05002905	0.66078000	11.49476962	0.04300000	2.40404000	0.31600000	1	0.76000000
499	7.80376982	5.66270000	1.77387689	5.93400000	56.50390000	1.32700000	1	6.79000000
500	0.70710678	1.90389000	2.27040466	2.09900000	14.52455000	0.43000000	1	2.29000000
501	1.91421356	−0.27920000	10.72505144	−0.54500000	4.48151000	0.55800000	1	−0.93000000
502	4.36640828	4.05810000	12.22761737	2.47200000	36.70150000	1.30400000	1	2.35000000
503	4.77128779	1.86257000	10.86468348	2.03400000	26.61871000	1.81400000	1	1.55000000
504	6.95690889	1.55730000	11.13648337	2.04900000	28.22458000	1.60300000	1	1.99000000
505	4.90559372	4.70880000	2.24541495	5.37400000	50.58369000	1.79800000	0	5.05000000
506	3.41421356	4.17690000	6.10387377	4.50100000	40.31129000	1.26200000	1	4.49000000

Table S3. *Cont.*

ID	*SP3*	*CrippenLogP*	*gmax*	*XLogP*	*DCW*	*MLFER.E*	*SubFP302*	$\log K_{ow}Epi$
507	3.81229215	2.05298000	12.16135818	2.27800000	30.34346000	0.78200000	1	1.67000000
508	3.60439768	2.16858000	11.24065193	2.43600000	22.52663000	1.21600000	1	1.97000000
509	4.67089426	2.94878000	11.54620748	3.15200000	27.13005000	1.21600000	1	2.95000000
510	6.04111925	1.92268000	12.08060234	1.07700000	12.86369000	0.66600000	1	1.38000000
511	3.12132034	3.43740000	12.42150720	2.62700000	30.81617000	0.86400000	1	3.14000000
512	5.02725307	3.32568000	11.45748437	2.26300000	26.70788000	1.23800000	1	3.15000000
513	6.60473761	4.56477000	11.77248759	4.59600000	35.25504000	1.76000000	1	3.22000000
514	6.84043987	4.20269000	11.83746536	4.49600000	35.03691000	1.76800000	1	3.27000000
515	5.56007941	2.95089000	11.51569107	3.58500000	32.46076000	1.65200000	1	2.48000000
516	7.08762781	4.21018000	12.24666745	4.91400000	41.39387000	1.65400000	1	3.33000000
517	4.54996918	2.65960000	11.33662210	2.03200000	27.25994000	1.14400000	1	2.58000000
518	5.71057106	6.01560000	2.32393004	5.14400000	61.53586000	2.06800000	0	6.34000000
519	5.77849536	3.74808000	12.12548973	3.94100000	35.49825000	1.20200000	1	3.37000000
520	2.54611208	0.78648000	10.41542559	1.04900000	10.28533000	0.74100000	1	0.61000000
521	6.93421579	3.17567000	12.30497780	3.05200000	26.55589000	2.32100000	1	2.24000000
522	4.13940532	3.36978000	10.86767920	2.40500000	28.94010000	1.16600000	1	3.04000000
523	5.56258335	2.60217000	11.41792423	1.66800000	24.04764000	1.95200000	1	2.65000000
524	6.08982602	4.56559000	12.58961845	4.21000000	33.71842000	1.29700000	1	4.15000000
525	6.08982602	4.56559000	12.58961845	4.21000000	33.71842000	1.29700000	1	4.15000000
526	6.90275936	2.00869000	11.58221829	1.81500000	30.01094000	1.85200000	1	2.73000000
527	7.34685469	5.22188000	12.17916528	4.20400000	43.18106000	1.43400000	1	4.81000000
528	4.82312892	2.64018000	11.37491300	1.47600000	25.55532000	1.15800000	1	2.11000000
529	6.29044696	3.04188000	11.64198476	1.73700000	26.62492000	1.51800000	1	3.22000000
530	4.41256506	2.29798000	11.00685744	1.33400000	23.25361000	1.18000000	1	1.90000000
531	3.47762381	2.28938000	10.88363883	1.77800000	22.87466000	1.15800000	1	1.82000000
532	4.69280295	0.95498000	11.76465278	1.12200000	23.76013000	1.15100000	1	1.49000000
533	7.32119623	5.78758000	6.58737654	6.75800000	55.06649000	2.44900000	1	6.34000000
534	4.63336239	2.74237000	8.90824035	0.78100000	22.34508000	1.98800000	1	2.51000000

Table S3. *Cont.*

ID	*SP3*	*CrippenLogP*	*gmax*	*XLogP*	*DCW*	*MLFER.E*	*SubFP302*	$\log K_{ow}Epi$
535	4.13940532	2.31740000	10.96856655	1.89000000	24.95824000	1.16600000	1	2.36000000
536	5.51601475	2.60471000	12.63999315	3.23100000	29.13895000	1.09300000	1	3.29000000
537	5.36593209	2.01088000	11.11271447	2.12900000	18.24967000	1.07100000	1	2.55000000
538	5.67658649	1.67762000	11.48659392	1.23100000	17.59858000	1.41200000	1	1.40000000
539	4.04669447	−0.14522000	11.41158211	0.37000000	11.43744000	0.99600000	1	−1.20000000
540	6.54749776	4.91838000	12.39928941	5.29100000	42.40337000	1.20200000	1	4.84000000
541	6.48005585	1.99028000	11.18937625	2.53400000	33.73211000	2.50000000	1	1.10000000
542	4.85138034	3.18298000	11.95331876	2.91900000	29.35207000	1.33400000	1	3.57000000
543	6.37972670	4.10279000	12.52791829	3.92700000	38.14019000	1.40100000	1	4.23000000
544	5.61856099	1.12789000	12.01761574	1.51700000	21.47269000	1.42500000	1	1.67000000
545	3.78467723	2.03728000	10.52769078	0.81600000	20.95191000	1.23100000	1	1.43000000
546	6.05155156	3.16679000	12.21882080	5.90900000	34.35140000	1.26500000	1	4.40000000
547	4.68297926	3.55840000	12.78100807	4.65000000	37.07167000	1.14200000	1	3.57000000
548	6.23531687	3.07228000	11.29899813	1.95500000	29.03425000	0.81100000	1	2.89000000
549	4.70689938	2.33278000	5.01483017	1.42800000	19.04569000	1.73700000	1	3.64000000
550	5.91371675	3.09491000	11.23850214	4.02200000	39.97467000	1.73000000	1	3.80000000
551	7.28762447	4.08699000	12.91688770	4.80200000	52.29475000	1.36900000	1	5.62000000
552	6.37867138	1.72249000	10.96750137	2.25400000	37.89448000	1.60600000	1	2.02000000
553	7.18844088	3.05367000	12.67242441	4.19200000	49.58499000	1.28200000	1	2.19000000
554	4.31195135	1.98678000	11.28585365	1.38000000	20.07924000	1.02300000	1	1.46000000
555	5.01917788	3.12859000	11.87423271	3.52000000	35.29191000	1.38400000	1	3.90000000
556	7.03829409	4.05199000	12.89331774	3.95700000	45.67539000	1.45100000	1	3.96000000
557	6.28090603	2.78261000	5.95148169	3.20300000	24.63235000	1.84500000	1	4.00000000
558	3.58154730	3.43159000	10.30933464	3.21300000	25.70830000	1.38000000	1	3.35000000
559	2.60815435	0.49389000	11.42505144	−0.26900000	7.40507000	0.59300000	1	−0.90000000
560	5.73664850	1.98512000	12.64336892	1.20200000	22.25084000	1.13500000	1	1.13000000
561	5.92637208	2.36711000	12.64505681	2.01000000	22.62668000	1.14600000	1	1.26000000
562	6.18146376	5.24698000	11.53498976	3.92300000	42.18757000	1.00200000	1	5.08000000

Table S3. *Cont.*

ID	*SP3*	*CrippenLogP*	*gmax*	*XLogP*	*DCW*	*MLFER.E*	*SubFP302*	$\log K_{ow}Epi$
563	5.76448110	6.01560000	2.43285322	5.14400000	61.53586000	2.06800000	0	6.34000000
564	6.90275936	3.91579000	12.85137330	4.82900000	51.30304000	1.30300000	1	5.07000000
565	6.52206541	4.29079000	11.28853316	6.89300000	36.78892000	1.39000000	1	5.15000000
566	6.65728680	4.53269000	11.44065476	5.13100000	40.90880000	1.46100000	1	5.80000000
567	4.98302034	2.14147000	11.37227749	1.33200000	26.37239000	1.27300000	1	1.78000000
568	4.67741012	3.10158000	11.34584199	3.43500000	26.38727000	0.99400000	1	2.84000000
569	5.72040601	3.65851000	12.12548973	3.79500000	35.49825000	1.20200000	1	3.37000000
570	4.84383758	4.70880000	2.24541495	5.16300000	50.58369000	1.79800000	0	5.05000000
571	6.72959710	7.32240000	2.53339807	5.33600000	72.48803000	2.33800000	0	7.62000000
572	6.93541370	3.77818000	13.39460800	4.68800000	38.04197000	1.68500000	1	3.59000000
573	5.31258805	5.36289000	6.45279331	5.83000000	47.95531000	1.91500000	1	5.65000000
574	6.14264985	4.12408000	5.94421265	3.05900000	34.05030000	1.49700000	1	4.10000000
575	5.59043127	6.01560000	2.43285322	5.14400000	61.53586000	2.06800000	0	6.34000000
576	6.16932307	7.97700000	2.59063310	7.36400000	55.91314000	3.37600000	0	9.10000000
577	4.11933192	2.03422000	11.34964097	2.68100000	32.19965000	1.01700000	1	2.48000000
578	6.91688183	3.00508000	12.35880921	2.31200000	41.20600000	2.00300000	1	2.85000000
579	5.24801249	5.36220000	2.40134431	5.25900000	56.05977000	1.93300000	0	5.69000000
580	5.22562352	5.36220000	2.40145062	5.25900000	56.05977000	1.93300000	0	5.69000000
581	6.16001419	6.66900000	2.51078532	5.24000000	67.01195000	2.20300000	0	6.98000000
582	6.42778838	6.66900000	2.45546597	5.24000000	67.01195000	2.20300000	0	6.98000000
583	7.26514550	7.32240000	2.42436858	5.33600000	72.48803000	2.33800000	0	7.62000000
584	6.61249576	3.70728000	12.34752630	4.12000000	38.42180000	1.22800000	1	3.62000000
585	6.57927691	3.49414000	11.27073980	5.68000000	36.08725000	1.41900000	1	4.82000000
586	5.35157341	5.28989000	12.78508561	4.04200000	40.13542000	1.61500000	1	4.82000000
587	5.61292987	0.65658000	11.72546674	2.60500000	29.33256000	1.46600000	1	1.44000000
588	5.25353557	2.78240000	4.16020576	2.41600000	32.89783000	2.04500000	0	2.48000000
589	5.38640489	3.66968000	5.94587449	3.26800000	35.36074000	1.18900000	1	3.71000000
590	7.54007881	4.80868000	11.68622094	4.32100000	44.02870000	1.90100000	1	4.15000000

Table S3. *Cont.*

ID	*SP3*	*CrippenLogP*	*gmax*	*XLogP*	*DCW*	*MLFER.E*	*SubFP302*	$\log K_{ow}Epi$
591	7.87338806	6.03858000	12.68125628	5.76000000	54.52870000	1.44800000	1	5.21000000
592	6.75451004	3.97778000	12.52893436	2.60300000	36.96533000	1.34000000	1	2.94000000
593	6.10669840	3.69941000	12.36035084	3.69500000	37.79996000	1.18700000	1	3.24000000
594	6.46329518	1.21978000	12.27335601	1.59200000	22.54924000	1.28800000	1	2.15000000
595	7.53203125	5.96147000	11.32518791	4.66100000	43.16619000	1.61900000	1	4.54000000
596	10.11207085	6.90588000	12.99428481	7.78200000	58.49378000	2.20800000	1	6.76000000
597	4.67089426	2.98027000	11.45611489	2.15100000	27.31547000	1.72900000	1	2.10000000
598	9.39123268	6.33007000	12.72429316	6.90900000	50.83505000	2.03400000	1	6.38000000
599	8.62395230	6.43467000	12.50609871	7.31300000	51.57718000	1.90500000	1	7.43000000
600	6.94574176	7.32240000	2.55779150	5.33600000	72.48803000	2.33800000	0	7.62000000
601	7.54625503	7.97580000	2.53398106	5.43200000	77.96412000	2.47300000	0	8.27000000
602	6.88235882	3.78918000	12.82659964	4.17200000	44.83653000	1.31100000	1	5.23000000
603	4.62899289	2.16879000	11.32930556	−0.58200000	11.88636000	1.16600000	0	0.04000000
604	4.30905462	3.23988000	10.31466534	1.51100000	38.17752000	1.20200000	1	2.53000000
605	4.60916222	2.18977000	11.30431453	1.64800000	22.34752000	0.77700000	1	1.51000000
606	5.09099026	3.93930000	12.25462354	3.28000000	34.80868000	1.31500000	1	2.46000000
607	6.62723179	2.09923000	12.30310941	2.33000000	30.80165000	1.10000000	1	1.70000000
608	4.56915739	4.63810000	5.88308728	4.50200000	45.87761000	1.44300000	1	5.18000000
609	6.30984143	1.84777000	11.70962195	2.70100000	27.34850000	1.28800000	1	1.91000000
610	8.50010191	4.12927000	12.92434131	7.91600000	66.20622000	1.74700000	1	4.48000000
611	8.29075687	3.26110000	11.65652935	4.43300000	45.65597000	2.28200000	1	3.62000000
612	7.83062486	3.71749000	6.39824043	3.16300000	46.28806000	1.55600000	1	4.13000000
613	6.89256502	3.93428000	12.68267570	4.19200000	50.11908000	1.24600000	1	3.45000000
614	9.46385528	4.94009000	14.18446576	6.03600000	65.33969000	1.81700000	1	6.09000000
615	4.01092395	3.62338000	11.24227183	4.34300000	27.19475000	0.89600000	1	3.72000000
616	7.44093622	2.64908000	12.36820358	2.36700000	23.85159000	2.25000000	1	2.26000000
617	3.35105733	0.82587000	5.37333711	−0.66900000	14.83900000	2.64300000	1	0.96000000
618	10.11207085	6.90588000	12.99428481	7.78200000	58.49378000	2.20800000	1	6.76000000

Table S3. *Cont.*

ID	*SP3*	*CrippenLogP*	*gmax*	*XLogP*	*DCW*	*MLFER.E*	*SubFP302*	$\log K_{ow}Epi$
619	8.72774028	6.24456000	11.57500169	4.57600000	40.58179000	1.98900000	1	4.95000000
620	10.42745602	7.68688000	13.05999023	9.23200000	62.55384000	2.78500000	1	7.56000000
621	7.66891985	4.70118000	12.43169867	2.91200000	43.73705000	1.99600000	1	4.13000000
622	9.94031461	6.46917000	14.18338486	6.86800000	51.42583000	1.91600000	1	5.74000000
623	8.64403359	5.89767000	12.51104599	6.03700000	50.95478000	1.03500000	1	5.34000000
624	10.74770672	6.85388000	13.01058240	7.95600000	62.17199000	1.91100000	1	6.56000000
625	4.14164776	1.77758000	10.61558883	2.89100000	22.58465000	1.16200000	1	2.26000000
626	6.37381015	3.82168000	12.47702483	4.31700000	32.92765000	0.99200000	1	3.43000000
627	8.08443988	2.26992000	12.53549131	2.91700000	26.23140000	2.17100000	1	1.71000000
628	8.52371897	1.59079000	12.55462711	2.04000000	21.80079000	2.16300000	1	2.00000000
629	9.03736329	6.04656000	11.59907141	4.49800000	42.75645000	2.17600000	1	4.35000000
630	8.11026415	1.57969000	14.19913313	3.73200000	39.94511000	1.62000000	1	2.52000000
631	5.04856315	2.79672000	11.44918096	2.48200000	26.58103000	1.32500000	1	3.37000000
632	5.16247589	3.66837000	10.62451929	3.26300000	28.70363000	1.72000000	1	2.76000000
633	3.51151749	1.54240000	12.69219152	1.23500000	17.77122000	0.96400000	1	1.45000000
634	3.51151749	2.16580000	10.49065866	1.71000000	19.89405000	1.39000000	1	2.14000000
635	8.33642620	4.23759000	12.59996336	4.13600000	45.87021000	1.98300000	1	5.57000000
636	6.69850831	4.20337000	11.06720499	4.54500000	33.99520000	1.50200000	1	4.24000000
637	8.27371897	1.65229000	12.46981230	1.15200000	18.92349000	2.14300000	1	1.27000000
638	7.13969043	1.03929000	12.00148998	1.04900000	22.78887000	1.46900000	1	1.57000000
639	8.79161231	4.99547000	12.61265558	4.22900000	42.30599000	1.25400000	1	3.98000000
640	10.45083594	6.25100000	12.86533484	9.56600000	75.63984000	1.71200000	1	8.15000000
641	7.98821335	3.62792000	6.41509897	5.31500000	40.34930000	1.04400000	1	4.55000000
642	8.62378535	2.75137000	12.66337991	2.88300000	29.27175000	2.31200000	1	2.29000000
643	10.75177939	6.69607000	12.89893895	7.07500000	52.15960000	1.60700000	1	6.85000000

Table S4. External validation criteria [22] of QSPR models in order to assure predictive capability: $1-R_0^2/R_{test}^2<0.1$ or $1-R_0^{'2}/R_{test}^2<0.1$; $0.85\le k\le 1.15$ or $0.85\le k^{'}\le 1.15$; $R_m^2>0.5$.

**Model**	R_{test}^2	k	R_0^2	$1-R_0^2/R_{test}^2$	$k^{'}$	$R_0^{'2}$	$1-R_0^{'2}/R_{test}^2$	R_m^2
Equation (1)	0.81	0.99	0.80	0.011	0.97	0.80	0.0096	0.73
Equation (2)	0.76	1.02	0.75	0.022	0.94	0.75	0.014	0.66
Equation (3)	0.84	0.98	0.84	0.0046	0.99	0.83	0.0096	0.79

Table S5. List of 64 structural attributes and their Correlation Weights *(CW)* involved in the best optimal descriptor obtained for the soil sorption coefficient.

**Structural Attribute**	***CW***
EC0-C...2...	−0.75233
EC0-C...3...	1.00094
EC0-C...4...	1.53053
EC0-F...1...	1.50152
EC0-H...1...	−0.18254
EC0-Br..1...	2.87376
EC0-Cl..1...	2.37291
EC0-N...2...	0.00219
EC0-N...3...	−0.75445
EC0-O...1...	0.12508
EC0-O...2...	−0.74928
EC0-P...4...	−0.75367
EC0-S...1...	4.24767
EC0-S...2...	2.75320
EC0-S...3...	−0.75460
EC0-S...4...	−0.75164
EC0-s...2...	1.50383
NNC-C...202.	−0.74836
NNC-C...211.	2.26082
NNC-C...303.	−0.75284
NNC-C...312.	−0.12066
NNC-C...321.	1.32745
NNC-C...330.	4.12048
NNC-C...404.	1.29582
NNC-C...413.	1.50017
NNC-C...422.	0.74549
NNC-F...101.	2.62383
NNC-F...110.	−0.75059
NNC-H...101.	−0.07978
NNC-H...110.	0.34269
NNC-Br..101.	3.12371
NNC-Cl..101.	1.50112
NNC-Cl..110.	3.26333
NNC-N...202.	1.50482
NNC-N...211.	4.01820
NNC-N...220.	0.49985
NNC-N...303.	−0.75467
NNC-N...312.	2.24966

Table S5. *Cont.*

Structural Attribute	*CW*
NNC-N...321.	0.93752
NNC-O...101.	0.24867
NNC-O...110.	−0.75205
NNC-O...202.	−0.43281
NNC-O...211.	−0.75099
NNC-O...220.	−0.25491
NNC-P...404.	−0.74691
NNC-P...413.	0.00444
NNC-S...101.	4.50159
NNC-S...202.	2.74528
NNC-S...211.	3.00164
NNC-S...303.	2.26953
NNC-S...312.	−0.75243
NNC-S...413.	−0.75330
NNC-s...220.	2.25354
NOSP00000000	4.50324
NOSP01000000	−0.74613
NOSP01010000	−0.75401
NOSP01100000	2.71627
NOSP01110000	4.50146
NOSP10000000	2.24712
NOSP10100000	3.74620
NOSP11000000	0.50479
NOSP11010000	−0.74649
NOSP11100000	−0.75472
NOSP11110000	−0.75304

Table S6. The best linear QSPR models with *d* descriptors obtained from a pool of 3492 geometry independent descriptors obtained from PaDEL and CORAL freewares.

d	Descriptors	R^2_{train}	R^2_{test}	RMS_{train}	RMS_{test}
1	*DCW*	0.87	0.76	0.44	0.60
2	*maxHBint2 DCW*	0.88	0.78	0.43	0.58
3	*maxHBint2 KRFPC2306 DCW*	0.89	0.78	0.40	0.58
4	*minHBint2 ExtFP394 KRFPC2306 DCW*	0.91	0.78	0.36	0.58
5	*minHBint2 FP541 ExtFP394 KRFPC2306 DCW*	0.91	0.77	0.36	0.59
6	*MATS4i minHBint2 FP541 ExtFP394 KRFPC2306 DCW*	0.93	0.77	0.33	0.59

Table S7. Experimental and predicted $\log K_{oc}$ values for 643 heterogeneous organic compounds. The last column includes the leverage values of compounds ($h^* = 0.129$) for Equation (3). Chemicals in the training set are reported in normal font and those in the prediction set in **Bold**.

ID	Chemical Compound	Chemical Abstracts Service Number	Experimental	Predicted Equation (1)	Predicted Equation (2)	Predicted Equation (3)	h_i
1	Formaldehyde	000050-00-0	**0.6**	0.46	0.25	0.98	0.00
2	4,4′-DDT	00005 0-29-3	5.3	5.12	5.61	4.73	0.05
3	Benzo[a]pyrene	000050-32-8	**5.8**	5.87	5.84	5.74	0.14
4	4-Methoxyacetanilide	000051-66-1	**1.4**	1.81	1.86	1.54	0.02
5	Trichlorfon	000052-68-6	**1.6**	1.35	2.06	0.97	0.02
6	Dibenz[a,h]anthracene	000053-70-3	**6.3**	6.36	6.20	6.10	0.15
7	Nicotine	000054-11-5	2	2.24	2.42	1.41	0.02
8	Benzamide	000055-21-0	**1.5**	1.23	1.72	1.31	0.02
9	Fenthion	000055-38-9	3.2	3.16	3.22	3.28	0.02
10	Tetrachloromethane	000056-23-5	**1.9**	2.27	2.50	2.14	0.01
11	Parathion(=ethyl parathion)	000056-38-2	**3.2**	2.87	2.64	3.08	0.02
12	3-Methylcholanthrene	000056-49-5	**6.1**	5.83	5.88	5.96	0.12
13	Diethylstilbestrol	000056-53-1	**4.1**	3.92	3.91	4.14	0.03
14	Benzo[a]anthracene	000056-55-3	**5.4**	5.37	5.09	5.09	0.10
15	Urea	000057-13-6	**1**	−0.33	0.46	0.24	0.02
16	Propyleneglycol	000057-55-6	**0.4**	0.27	0.58	0.19	0.04
17	7,12-Dimethylbenz[a]anthracene	000057-97-6	**5.4**	5.68	5.69	5.63	0.10
18	Lindane	000058-89-9	**3**	3.65	4.09	3.36	0.03
19	2,3,4,6-Tetrachlorophenol	000058-90-2	**3.4**	2.72	2.81	3.38	0.03
20	p-Phenylazoaniline	000060-09-3	**2.8**	3.36	3.29	2.90	0.02
21	4-Dimethylaminoazobenzene	000060-11-7	**3.9**	3.86	3.62	3.41	0.02
22	2-Phenylethanol	000060-12-8	**1.5**	1.71	1.57	1.61	0.02
23	Dimethoate	000060-51-5	**1.1**	1.24	1.62	1.44	0.03
24	Dieldrin	000060-57-1	**4.4**	4.28	4.64	4.47	0.06
25	Amitrol	000061-82-5	1.6	0.75	1.12	1.22	0.05

Table S7. *Cont.*

ID	Chemical Compound	Chemical Abstracts Service Number	Experimental	Predicted Equation (1)	Predicted Equation (2)	Predicted Equation (3)	h_i
26	4-Nitrobenzoic acid	000062-23-7	**1.5**	1.55	1.67	1.81	0.02
27	Aniline	000062-53-3	**1.4**	2.05	1.63	1.80	0.01
28	Thioacetamide	000062-55-5	**0.8**	0.76	1.15	0.78	0.03
29	Thiourea	000062-56-6	**0.9**	0.36	1.05	0.67	0.05
30	Dichlorvos	000062-73-7	**1.7**	1.37	1.10	0.96	0.02
31	Carbaryl	000063-25-2	**2.4**	2.54	2.50	2.44	0.02
32	3-Methylphenylurea	000063-99-0	**1.6**	1.43	1.62	1.61	0.02
33	Phenylurea	000064-10-8	**1.4**	1.33	1.46	1.35	0.03
34	Ethanol	000064-17-5	**0.2**	0.47	0.52	0.77	0.00
35	Acetic Acid	000064-19-7	**0**	0.44	0.26	0.85	0.00
36	Benzoic Acid	000065-85-0	1.5	1.54	1.43	1.72	0.02
37	Methanol	000067-56-1	**0.4**	0.34	0.36	0.54	0.00
38	Trichloromethane	000067-66-3	**1.7**	1.98	2.10	1.66	0.00
39	Hexachloroethane	000067-72-1	**3.3**	3.19	3.16	2.75	0.03
40	1-Propanol	000071-23-8	**0.5**	0.71	0.69	0.65	0.03
41	1-Butanol	000071-36-3	0.5	0.94	0.86	0.88	0.03
42	1-Pentanol	000071-41-0	**0.7**	1.18	1.02	1.12	0.03
43	Benzene	000071-43-2	**1.9**	2.42	1.73	2.07	0.01
44	1,1,1-Trichloroethane	000071-55-6	**2**	2.06	2.25	2.21	0.02
45	Endrin	000072-20-8	**4.1**	4.28	4.50	4.47	0.06
46	Methoxychlor	000072-43-5	**4.9**	4.60	4.02	4.06	0.03
47	4,4-DDD	000072-54-8	**4.8**	4.82	4.75	4.24	0.03
48	4,4′-DDE	000072-55-9	4.8	4.88	4.37	4.42	0.03
49	Methyl bromide	000074-83-9	1.3	1.49	1.09	1.49	0.00
50	Dichloromethane	000075-09-2	**1.4**	1.70	1.69	1.53	0.00
51	Ethylene Oxide	000075-21-8	**0.3**	0.74	0.56	0.85	0.00
52	Tribromomethane	000075-25-2	**2.2**	2.26	1.49	2.10	0.01

Table S7. *Cont.*

ID	Chemical Compound	Chemical Abstracts Service Number	Experimental	Predicted Equation (1)	Predicted Equation (2)	Predicted Equation (3)	h_i
53	Bromodichloromethane	000075-27-4	1.8	2.07	1.89	1.81	0.00
54	1,1-Dichloroethane	000075-34-3	**1.5**	1.79	1.85	1.72	0.01
55	1,1-Dichloroethene	000075-35-4	**1.8**	1.98	1.41	1.93	0.01
56	Trichlorofluoromethane	000075-69-4	**2.2**	1.21	2.14	1.87	0.01
57	Dalapon	000075-99-0	0.4	1.14	1.44	1.39	0.02
58	Chloropicrin	000076-06-2	**1.8**	1.27	1.96	1.33	0.02
59	Heptachlor	000076-44-8	**4**	4.60	4.06	4.59	0.06
60	Hexachlorocyclopentadiene	000077-47-4	**3.2**	3.76	2.97	3.71	0.04
61	Tribuphos	000078-48-8	**3.7**	3.57	3.73	3.83	0.05
62	Isophorone	000078-59-1	**1.4**	1.70	1.30	2.28	0.01
63	1,2-Dichloropropane	000078-87-5	**1.7**	1.99	1.90	1.61	0.03
64	1,1,2-Trichloroethane	000079-00-5	1.8	2.13	2.15	1.57	0.02
65	Trichloroethene	000079-01-6	**2**	2.25	1.81	2.24	0.01
66	1,1,2,2-Tetrachloroethane	000079-34-5	**1.9**	2.48	2.46	1.73	0.02
67	Warfarin	000081-81-2	**3**	3.64	3.36	2.69	0.04
68	1-Amino-2-methyl-9,10-Anthracenedione	000082-28-0	**3.9**	2.79	3.10	3.92	0.05
69	Pentachloronitrobenzene	000082-68-8	**4.6**	3.22	3.60	3.74	0.02
70	Acenaphthene	000083-32-9	3.9	3.72	3.19	3.68	0.04
71	9,10-Anthracenedione	000084-65-1	**3.6**	2.62	2.79	3.40	0.04
72	Diethyl phthalate	000084-66-2	**1.8**	2.10	2.08	2.08	0.02
73	Diisobutyl phthalate	000084-69-5	**3.1**	2.82	2.70	2.93	0.04
74	Di-n-butyl phthalate	000084-74-2	**3.1**	3.02	2.70	3.02	0.05
75	Di-n-hexyl phthalate	000084-75-3	**4.7**	4.00	3.49	3.96	0.10
76	Phenanthrene	000085-01-8	**4.3**	4.39	3.97	4.09	0.05
77	Benzo[f]quinoline	000085-02-9	**4.8**	3.67	3.51	3.58	0.05
78	Pentabromoethylbenzene	000085-22-3	**4.9**	4.60	3.05	5.27	0.06
79	Fenac	000085-34-7	1.6	2.37	2.67	2.68	0.02

Table S7. *Cont.*

ID	Chemical Compound	Chemical Abstracts Service Number	Experimental	Predicted Equation (1)	Predicted Equation (2)	Predicted Equation (3)	h_i
80	1,3-Isobenzofurandione	000085-44-9	**1.6**	1.53	1.53	2.42	0.02
81	n-Butyl benzyl phthalate	000085-68-7	**3.7**	3.53	3.31	3.51	0.03
82	2-Butoxy-2-oxoethyl butyl phthalate	000085-70-1	3.7	3.03	3.03	2.83	0.03
83	N-Nitrosodiphenylamine	000086-30-6	**3.1**	2.91	2.66	2.70	0.01
84	Azinphos methyl	000086-50-0	**2.6**	2.69	3.75	2.78	0.03
85	Fluorene	000086-73-7	**3.7**	3.95	3.32	3.70	0.04
86	Carbazole	000086-74-8	3.7	3.41	3.30	3.45	0.05
87	1-Naphthalene acetamide	000086-86-2	2	2.30	2.72	2.22	0.03
88	1-Naphthalene acetic acid	000086-87-3	**2.3**	2.62	2.42	2.52	0.02
89	1,2,3-Trichlorobenzene	000087-61-6	**3.3**	3.11	2.94	3.24	0.03
90	Pentachlorophenol	000087-86-5	**3.3**	3.05	3.22	3.78	0.04
91	2,4,6-Trichlorophenol	000088-06-2	**3**	2.37	2.41	3.00	0.02
92	2-Nitrophenol	000088-75-5	**2.1**	1.73	1.45	1.94	0.02
93	Dinoseb	000088-85-7	**2.4**	2.91	2.30	2.95	0.02
94	Phthalic acid	000088-99-3	**1.1**	1.45	1.40	1.41	0.02
95	2-Methoxyphenol	000090-05-1	**1.6**	1.62	1.23	1.47	0.02
96	1-Methylnaphthalene	000090-12-0	**3.4**	3.55	3.30	3.35	0.03
97	1-Naphthol	000090-15-3	3	2.49	2.32	2.95	0.03
98	4,4Bis(dimethylamino)benzophenone	000090-94-8	**2.2**	2.08	3.17	2.18	0.05
99	Diphenylmethanol	000091-01-0	**2.3**	2.70	2.88	2.52	0.01
100	1,2-Dimethoxybenzene	000091-16-7	**2**	2.24	1.40	1.57	0.02
101	Naphthalene	000091-20-3	**3.1**	3.40	2.85	3.08	0.02
102	Quinoline	000091-22-5	**3**	2.69	2.39	2.57	0.02
103	2-Methylnaphthalene	000091-57-6	**3.5**	3.52	3.30	3.35	0.03
104	*N*,*N*-Diethyl Aniline	000091-66-7	**2.4**	2.39	1.96	1.94	0.01
105	Methapyrilene	000091-80-5	**2.9**	2.92	2.80	2.66	0.02
106	3,3′-Dichlorobenzidine	000091-94-1	**4.4**	3.67	3.97	3.67	0.07

Table S7. *Cont.*

ID	Chemical Compound	Chemical Abstracts Service Number	Experimental	Predicted Equation (1)	Predicted Equation (2)	Predicted Equation (3)	h_i
107	Tetracene	000092-24-0	**5.8**	5.36	5.09	5.09	0.10
108	Biphenyl	000092-52-4	**3.3**	3.88	3.22	3.44	0.03
109	Phenazine	000092-82-0	**3.4**	3.38	2.86	3.07	0.06
110	Benzidine	000092-87-5	**3.5**	3.12	3.17	2.89	0.07
111	4-Acetylbiphenyl	000092-91-1	**3.2**	3.13	3.21	3.02	0.02
112	2-Acetonaphthalene	000093-08-3	**2.9**	2.65	2.84	2.65	0.02
113	Methyl benzoate	000093-58-3	**2.1**	1.66	1.60	1.66	0.02
114	Silvex	000093-72-1	**2.5**	2.78	2.63	2.85	0.02
115	2,4,5-T acid	000093-76-5	**1.9**	2.48	2.54	2.66	0.01
116	Ethyl benzoate	000093-89-0	**2.3**	1.84	1.71	1.90	0.02
117	Phenyl Benzoate	000093-99-2	**3.2**	2.89	2.40	2.62	0.01
118	Ethyl 4-methylbenzoate	000094-08-6	**2.6**	1.96	1.81	2.16	0.02
119	Safrole	000094-59-7	**2.8**	2.67	1.42	2.64	0.02
120	MCPA	000094-74-6	**1.7**	2.01	2.03	2.15	0.01
121	2,4-Dichlorophenoxy acetic acid	000094-75-7	**1.5**	2.17	2.14	2.27	0.01
122	Benzotriazole	000095-14-7	**1.7**	2.10	2.52	2.12	0.03
123	Benzo[b]thiophene	000095-15-8	**3.5**	2.94	2.45	2.98	0.02
124	1,2-Dimethylbenzene	000095-47-6	**2.4**	2.71	2.34	2.60	0.02
125	*o*-cresol	000095-48-7	**1.3**	1.72	1.36	2.21	0.01
126	2-Chlorotoluene	000095-49-8	**2.6**	2.65	2.29	2.72	0.02
127	1,2-Dichlorobenzene	000095-50-1	**2.5**	2.80	2.54	2.85	0.02
128	2-Chlorophenol	000095-57-8	**2.2**	1.85	1.61	2.27	0.01
129	1,3,4-Trimethylbenzene	000095-63-6	**3.6**	2.82	2.49	2.87	0.03
130	3,4-Dichloroaniline	000095-76-1	**2.3**	2.60	2.43	2.58	0.02
131	3,4-Dichlorophenol	000095-77-2	**3.1**	2.11	2.01	2.72	0.02
132	1,2,4,5-Tetramethylbenzene	000095-93-2	**3.1**	2.98	2.64	3.14	0.04
133	1,2,4,5-Tetrachlorobenzene	000095-94-3	**3.2**	3.36	3.34	3.63	0.04

Table S7. *Cont.*

ID	Chemical Compound	Chemical Abstracts Service Number	Experimental	Predicted Equation (1)	Predicted Equation (2)	Predicted Equation (3)	h_i
134	2,4,5-Trichlorophenol	000095-95-4	3	2.40	2.41	3.06	0.02
135	1,2-Dibromo-3-chloropropane	000096-12-8	2	2.47	2.53	2.11	0.02
136	3-Trifluoromethylaniline	000098-16-8	**2.4**	1.70	2.21	1.72	0.02
137	α-Methylbenzyl alcohol	000098-85-1	**1.5**	1.70	1.81	1.56	0.02
138	Acetophenone	000098-86-2	**1.6**	1.70	1.72	1.64	0.02
139	Nitrobenzene	000098-95-3	**2.1**	1.80	1.59	1.79	0.02
140	Amino-3-nitrobenzene	000099-09-2	**1.7**	1.52	1.65	1.79	0.02
141	Dichloran	000099-30-9	**3.4**	2.13	2.46	2.57	0.02
142	3,5-Dinitrobenzoic acid	000099-34-3	**1.9**	1.61	1.72	1.89	0.02
143	Trinitrobenzol	000099-35-4	**1.3**	1.93	1.90	1.96	0.03
144	3,4-Dichloronitrobenzene	000099-54-7	2.5	2.33	2.40	2.57	0.01
145	m-Dinitrobenzene	000099-65-0	**1.6**	1.85	1.74	1.87	0.02
146	Ethyl 4-nitrobenzoate	000099-77-4	**2.5**	1.84	1.90	1.98	0.01
147	4-Methylbenzoic acid	000099-94-5	**1.8**	1.67	1.58	1.99	0.02
148	4-Hydroxybenzoic Acid	000099-96-7	**1.4**	1.27	1.18	1.60	0.02
149	Amino-4-nitrobenzene	000100-01-6	**1.9**	1.54	1.65	1.79	0.02
150	4-Nitrophenol	000100-02-7	**2.1**	1.59	1.45	1.94	0.02
151	Ethylbenzene	000100-41-4	**2.3**	2.86	2.05	2.21	0.02
152	Styrene	000100-42-5	**3**	2.69	1.75	2.22	0.02
153	Benzyl alcohol	000100-51-6	**0.7**	1.45	1.66	1.38	0.02
154	3-Cyanopyridine	000100-54-9	**1.7**	1.26	1.31	1.36	0.01
155	*N*-methylaniline	000100-61-8	2.3	2.21	1.80	1.65	0.02
156	Anisole	000100-66-3	**1.5**	2.21	1.37	1.76	0.02
157	Anilazine	000101-05-3	**3.2**	3.62	2.94	3.54	0.04
158	Chlorpropham	000101-21-3	**2.7**	2.37	2.37	2.55	0.02
159	Fenuron	000101-42-8	**1.4**	1.72	1.80	1.62	0.02
160	4,4′-Methylenebis(*N*,*N*-Dimethylaniline)	000101-61-1	**4**	3.87	3.56	3.53	0.02

Table S7. *Cont.*

ID	Chemical Compound	Chemical Abstracts Service Number	Experimental	Predicted Equation (1)	Predicted Equation (2)	Predicted Equation (3)	h_i
161	4,4′Methylene dianiline	000101-77-9	**2**	3.15	2.89	2.51	0.03
162	Diphenyl ether	000101-84-8	**3.3**	3.41	2.35	3.09	0.02
163	Ethyl Phenylacetate	000101-97-3	1.9	1.97	1.96	2.02	0.02
164	Ethyl-*N*-phenylcarbamate	000101-99-5	**1.8**	1.96	1.72	1.97	0.02
165	1,3,5-Triethylbenzene	000102-25-0	**4.1**	3.72	2.70	3.22	0.06
166	Bis(2-ethylhexyl) adipate	000103-23-1	**4.2**	4.40	3.89	4.31	0.24
167	Azobenzene	000103-33-3	**3.2**	3.74	3.23	3.18	0.02
168	Propylbenzene	000103-65-1	**2.9**	3.08	2.25	2.45	0.03
169	2-Pyridineethanol	000103-74-2	**1.5**	1.20	1.21	1.03	0.02
170	Phenylacetic Acid	000103-82-2	**1.5**	1.66	1.62	1.51	0.02
171	Acetanilide	000103-84-4	**1.4**	1.77	1.56	1.48	0.02
172	4-Bromoacetanilide	000103-88-8	**2**	2.10	1.91	2.10	0.02
173	n-Buthylbenzene	000104-51-8	**3.4**	3.34	2.70	2.68	0.04
174	Ethyl heptanoate	000106-30-9	**2.6**	1.94	1.56	2.00	0.06
175	Ethyl octanoate	000106-32-1	**3**	2.19	1.73	2.23	0.07
176	4-Bromoaniline	000106-40-1	**2**	2.38	1.83	2.41	0.02
177	4-Bromophenol	000106-41-2	**2.4**	1.91	1.41	2.55	0.02
178	1,4-Dimethylbenzene	000106-42-3	**2.5**	2.67	2.04	2.60	0.02
179	4-Methylphenol	000106-44-5	**2.2**	1.71	1.36	2.21	0.01
180	1,4-Dichlorobenzene	000106-46-7	**2.5**	2.76	2.54	2.85	0.02
181	4-Chloroaniline	000106-47-8	**2**	2.30	2.03	2.18	0.01
182	4-Chlorophenol	000106-48-9	**1.9**	1.82	1.61	2.33	0.01
183	4-Methylaniline	000106-49-0	**1.9**	2.18	1.78	2.06	0.01
184	Epichlorohydrin	000106-89-8	**1**	1.19	1.18	0.88	0.02
185	1,2-Dibromoethane	000106-93-4	**1.7**	2.03	1.70	1.72	0.02
186	2-Propenal	000107-02-8	−0.3	0.65	0.43	0.63	0.03
187	1,2-Dichloroethane	000107-06-2	**1.5**	1.84	1.75	1.42	0.03

Table S7. *Cont.*

ID	Chemical Compound	Chemical Abstracts Service Number	Experimental	Predicted Equation (1)	Predicted Equation (2)	Predicted Equation (3)	h_i
188	1,3-Dimethylbenzene	000108-38-3	**2.8**	2.65	2.04	2.60	0.02
189	m-Cresol	000108-39-4	1.5	1.68	1.36	2.21	0.01
190	3-Chlorophenol	000108-43-0	**2.2**	1.80	1.61	2.33	0.01
191	3-Methylaniline	000108-44-1	**1.7**	2.16	1.78	2.06	0.01
192	3-Hydroxyphenol	000108-46-3	**1**	1.36	1.06	1.81	0.01
193	Bis(2-chloroisopropyl)ether	000108-60-1	**1.7**	2.13	1.88	2.31	0.05
194	1,3,5-Trimethylbenzene	000108-67-8	**2.8**	2.74	2.19	2.87	0.03
195	3,5-Dimethylphenol	000108-68-9	**2.8**	1.76	1.51	2.48	0.01
196	1,3,5-Trichlorobenzene	000108-70-3	**2.9**	2.98	2.94	3.24	0.03
197	Bromobenzene	000108-86-1	**2.3**	2.60	1.93	2.68	0.02
198	Toluene	000108-88-3	**2.2**	2.54	1.89	2.34	0.01
199	Chlorobenzene	000108-90-7	**2.4**	2.52	2.13	2.46	0.01
200	Phenol	000108-95-2	**1.4**	1.58	1.21	1.94	0.01
201	n-Butylamine	000109-73-9	**1.9**	1.18	1.28	0.89	0.03
202	1-Hexanol	000111-27-3	1	1.42	1.19	1.35	0.03
203	Bis(2-chloroethyl)ether	000111-44-4	**1.5**	1.78	1.56	1.20	0.03
204	1-Heptanol	000111-70-6	**1.1**	1.67	1.36	1.59	0.03
205	1-Octanol	000111-87-5	**1.6**	1.91	1.53	1.83	0.04
206	1-Decanol	000112-30-1	2.6	2.41	1.87	2.30	0.06
207	1-Dodecanol	000112-53-8	**3.5**	2.91	2.21	2.77	0.08
208	Propoxur	000114-26-1	1.5	2.04	1.85	1.78	0.02
209	2-Chlorophenylurea	000114-38-5	**1.6**	1.58	1.82	1.73	0.02
210	Chlorendic acid	000115-28-6	**2.8**	2.86	3.08	2.79	0.01
211	Endosulfan	000115-29-7	**4.1**	3.02	3.76	3.55	0.04
212	Dicofol	000115-32-2	**3.7**	3.92	4.68	4.36	0.03
213	Fensulfothion	000115-90-2	**2.5**	2.45	2.66	2.47	0.02
214	Aldicarb	000116-06-3	**1.6**	1.42	1.49	1.44	0.02

Table S7. *Cont.*

ID	Chemical Compound	Chemical Abstracts Service Number	Experimental	Predicted Equation (1)	Predicted Equation (2)	Predicted Equation (3)	h_i
215	2,3,5,6-Tetrachloronitrobenzene	000117-18-0	**4.1**	2.88	3.20	3.35	0.02
216	Di-2-ethylhexyl phthalate	000117-81-7	5	4.92	4.48	4.82	0.18
217	bis(n-octyl) Phthalate	000117-84-0	**4.4**	5.00	4.42	4.91	0.18
218	Hexachlorobenzene	000118-74-1	**3.8**	4.12	4.15	4.41	0.06
219	Chloranil	000118-75-2	**2.3**	2.13	1.77	2.59	0.02
220	2,4,6-Trinitrotoluene	000118-96-7	**2.7**	2.21	2.05	2.22	0.02
221	Benzophenone	000119-61-9	2.7	2.73	2.96	2.73	0.01
222	2,2′-Biquinoline	000119-91-5	**4**	4.70	4.34	4.45	0.11
223	Anthracene	000120-12-7	**4.3**	4.38	3.97	4.09	0.05
224	Dichloroprop	000120-36-5	**3**	2.47	2.31	2.46	0.02
225	Ethyl 4-hydroxybenzoate	000120-47-8	**2.2**	1.57	1.63	2.08	0.02
226	Catechol	000120-80-9	**2.1**	1.49	1.06	1.77	0.01
227	1,2,4-Trichlorobenzene	000120-82-1	**3.1**	3.06	2.94	3.24	0.03
228	2,4-Dichlorophenol	000120-83-2	**2.8**	2.10	2.01	2.66	0.02
229	*N*,*N*-Dimethylaniline	000121-69-7	2.3	2.39	3.56	1.94	0.01
230	Malathion	000121-75-5	**2.7**	2.00	2.61	2.07	0.01
231	3,5-Dinitrobenzamide	000121-81-3	2.3	1.30	2.03	1.47	0.05
232	Fenitrothion	000122-14-5	**2.6**	2.60	2.19	2.87	0.01
233	3-Nitroacetanilide	000122-28-1	**1.9**	1.83	1.71	1.91	0.02
234	Simazine	000122-34-9	**2.1**	2.26	1.87	2.63	0.03
235	Diphenylamine	000122-39-4	**2.8**	3.61	2.63	2.94	0.02
236	Propham	000122-42-9	1.8	2.14	2.00	2.16	0.02
237	1,2-Diphenylhydrazine	000122-66-7	**3**	3.71	2.82	2.83	0.02
238	Maleic hydrazine	000123-33-1	**0.5**	0.35	1.47	0.76	0.03
239	Ethyl hexanoate	000123-66-0	**2.1**	1.70	1.27	1.76	0.05
240	1,4-Dioxane	000123-91-1	**1.2**	0.95	0.81	0.73	0.00
241	Chlorodibromomethane	000124-48-1	**1.9**	2.17	1.69	1.96	0.01

Table S7. *Cont.*

ID	Chemical Compound	Chemical Abstracts Service Number	Experimental	Predicted Equation (1)	Predicted Equation (2)	Predicted Equation (3)	h_i
242	Tetrachloroethene	000127-18-4	**2.5**	2.62	2.42	2.56	0.02
243	Dibenzo[a,h]pyrene-7,14-dione	000128-66-5	**4.3**	4.86	5.78	6.06	0.18
244	Pyrene	000129-00-0	**4.8**	4.89	4.72	4.73	0.09
245	Dimethyl phthalate	000131-11-3	**2**	1.73	1.74	1.61	0.02
246	Dibenzofuran	000132-64-9	**3.9**	3.31	3.19	3.62	0.04
247	Dibenzothiophene	000132-65-0	**4.1**	3.83	3.47	3.99	0.05
248	Captan	000133-06-2	**2.3**	2.32	2.81	2.57	0.02
249	Folpet	000133-07-3	**3.3**	2.62	3.27	2.81	0.02
250	Chlorambed	000133-90-4	**1.4**	1.76	2.28	2.07	0.02
251	1-Aminonaphthalene	000134-32-7	**3.5**	3.00	2.74	2.80	0.04
252	Thiram	000137-26-8	2.8	2.42	3.09	2.39	0.04
253	Propazine	000139-40-2	**2.3**	2.67	2.21	3.02	0.02
254	4,4′-Thiodianiline	000139-65-1	**2**	3.08	3.07	2.84	0.04
255	Dicrotophos(cis)	000141-66-2	**1.7**	1.04	0.61	0.63	0.03
256	Hexanoic Acid	000142-62-1	**1.5**	1.39	0.93	1.43	0.03
257	1-Nonanol	000143-08-8	**1.9**	2.16	1.70	2.06	0.05
258	Chlordecone	000143-50-0	**4.2**	4.56	5.32	4.05	0.07
259	Endothal	000145-73-3	**2.1**	0.95	1.17	1.65	0.02
260	Thiabendazole	000148-79-8	**3.2**	3.03	2.99	2.90	0.06
261	2-Benzothiazolethiol	000149-30-4	**2.3**	2.57	2.57	2.77	0.06
262	4-Aminobenzoic acid	000150-13-0	**2.1**	1.22	1.58	1.45	0.02
263	3-Methoxyphenol	000150-19-6	**1.5**	1.59	1.23	1.63	0.02
264	Monuron	000150-68-5	**2**	1.96	2.15	2.02	0.01
265	4-Methoxyphenol	000150-76-5	**1.8**	1.61	1.23	1.63	0.02
266	Aziridine	000151-56-4	**0.8**	0.72	0.98	0.82	0.01
267	trans-1,2-Dichloroethene	000156-60-5	**1.8**	1.98	1.41	1.93	0.01
268	Dibenzo[a,i]pyrene	000189-55-9	**5.7**	6.85	6.95	6.74	0.21

Table S7. *Cont.*

ID	Chemical Compound	Chemical Abstracts Service Number	Experimental	Predicted Equation (1)	Predicted Equation (2)	Predicted Equation (3)	h_i
269	Benzo[g,h,i]perylene	000191-24-2	**5.4**	6.38	6.59	6.38	0.20
270	Benzo[e]pyrene	000192-97-2	**6.1**	5.87	5.84	5.74	0.14
271	Indeno[1,2,3-cd]pyrene	000193-39-5	**6.3**	6.37	6.59	6.38	0.20
272	*7H*-Dibenzo[c,g]carbazole	000194-59-2	**6**	5.36	6.02	5.46	0.14
273	Perylene	000198-55-0	**5.5**	5.87	5.84	5.74	0.14
274	Benzo[b]fluoranthene	000205-99-2	**5.4**	5.87	5.84	5.74	0.14
275	Fluoranthene	000206-44-0	**4.7**	4.89	4.72	4.73	0.09
276	Benzo[k]fluoranthene	000207-08-9	**5**	5.87	5.84	5.74	0.14
277	Acenaphthylene	000208-96-8	**3.8**	3.79	2.92	3.66	0.04
278	Chrysene	000218-01-9	5.5	5.38	5.09	5.09	0.10
279	Dibenz[a,j]anthracene	000224-41-9	6	6.35	6.20	6.10	0.15
280	Benz[a]acridine	000225-11-6	4.4	4.58	4.53	4.58	0.10
281	Benzo[a]fluorene	000238-84-6	**5.5**	4.94	4.44	4.71	0.07
282	13H-Dibenzo[a,i]carbazole	000239-64-5	**6**	5.30	5.53	5.46	0.14
283	Acridine	000260-94-6	**4.1**	3.60	3.41	3.58	0.05
284	Parathion methyl	000298-00-0	**3**	2.51	2.04	2.61	0.02
285	Phorate	000298-02-2	3.1	2.81	2.93	2.87	0.02
286	Disulfoton	000298-04-4	**3.1**	2.92	3.10	3.08	0.02
287	Naled	000300-76-5	2.2	1.98	1.85	1.69	0.02
288	Oxydemeton-Methyl	000301-12-2	**1.1**	1.23	1.95	0.42	0.05
289	Aldrin	000309-00-2	**4.4**	4.87	4.32	5.06	0.08
290	Bromacil	000314-40-9	**1.7**	1.69	2.11	1.94	0.02
291	Isocil	000314-42-1	**2.1**	1.44	1.94	1.71	0.02
292	alpha-HCH	000319-84-6	3.3	3.65	3.85	3.36	0.03
293	beta-HCH	000319-85-7	**3.4**	3.65	3.85	3.36	0.03
294	3-(3-Fluorophenyl)-1,1-dimethylurea	000330-39-2	**1.7**	1.64	1.80	1.65	0.02
295	Diuron	000330-54-1	2.5	2.27	2.56	2.40	0.01

Table S7. *Cont.*

ID	Chemical Compound	Chemical Abstracts Service Number	Experimental	Predicted Equation (1)	Predicted Equation (2)	Predicted Equation (3)	h_i
296	Linuron	000330-55-2	**2.8**	2.31	2.78	2.40	0.01
297	3-(4-Fluorophenyl)-1,1-dimethylurea	000332-33-2	1.4	1.22	1.51	1.37	0.02
298	Diazinon	000333-41-5	**2.8**	3.45	2.70	2.94	0.02
299	Mevinphos(trans)	000338-45-4	**2**	1.15	0.48	0.91	0.02
300	3-Fluoroacetanilide	000351-28-0	1.6	1.65	1.76	1.51	0.02
301	3-Trifluoromethylacetanilide	000351-36-0	**1.8**	2.20	2.43	1.77	0.02
302	4-Fluoroacetanilide	000351-83-7	**1.5**	1.68	1.76	1.51	0.02
303	2,2′-Bipyridine	000366-18-7	**1.6**	2.72	2.30	2.27	0.03
304	Auramine	000492-80-8	3.3	3.29	3.69	3.04	0.03
305	Indane	000496-11-7	**3.6**	2.99	2.53	2.91	0.02
306	Chlorobenzilate	000510-15-6	**3.4**	3.11	3.56	3.34	0.02
307	1,2,3-Trimethylbenzene	000526-73-8	2.8	2.87	2.19	2.87	0.03
308	Pentachloroaniline	000527-20-8	**4.6**	3.56	3.64	3.75	0.04
309	3,4-Dinitrobenzoic Acid	000528-45-0	**1.5**	1.62	1.83	1.89	0.02
310	2-Chloroacetanilide	000533-17-5	**1.6**	2.03	2.12	1.60	0.02
311	3-Methylacetanilide	000537-92-8	**1.5**	1.88	1.85	1.75	0.02
312	Ethyl pentanoate	000539-82-2	2	1.46	1.10	1.53	0.04
313	1,3-Dichlorobenzene	000541-73-1	2.5	2.73	2.54	2.85	0.02
314	1,3-Dichloropropene	000542-75-6	**1.5**	2.00	1.61	1.72	0.03
315	2,4-Dichloroaniline	000554-00-7	2.7	2.59	2.43	2.58	0.02
316	3-Nitrophenol	000554-84-7	**1.7**	1.58	1.45	1.94	0.02
317	Neburon	000555-37-3	**3.4**	2.94	3.16	3.11	0.02
318	Methyl Isothiocyanate	000556-61-6	**1**	1.14	1.23	1.72	0.00
319	Ethion	000563-12-2	**4.1**	3.60	3.60	3.88	0.02
320	2,3-Dichlorophenol	000576-24-9	**2.7**	2.14	2.01	2.66	0.02
321	2,3-dimethylnaphthalene	000581-40-8	**4.1**	3.68	3.45	3.61	0.03
322	4-Bromonitrobenzene	000586-78-7	**2.4**	2.12	1.79	2.40	0.01

Table S7. *Cont.*

ID	Chemical Compound	Chemical Abstracts Service Number	Experimental	Predicted Equation (1)	Predicted Equation (2)	Predicted Equation (3)	h_i
323	3-(3-Chlorophenyl)-1,1-dimethylurea	000587-34-8	**1.8**	1.55	1.87	1.73	0.02
324	*o*-Chloroacetanilide	000587-65-5	**1.6**	2.03	2.12	1.60	0.02
325	3-Chloroacetanilide	000588-07-8	**1.9**	2.00	2.12	1.87	0.02
326	3,5-Dichlorophenol	000591-35-5	**2.8**	2.03	2.01	2.72	0.02
327	Iodobenzene	000591-50-4	**3.1**	2.58	1.72	3.02	0.02
328	Dibromodichloromethane	000594-18-3	**1.9**	2.46	2.09	2.44	0.01
329	Trichloroacetamide	000594-65-0	**1**	0.91	1.73	1.16	0.02
330	Methyl-urea	000598-50-5	**1.8**	−0.06	0.63	0.07	0.04
331	2,6-Dichloroaniline	000608-31-1	**3.3**	2.62	2.43	2.58	0.02
332	Pentachlorobenzene	000608-93-5	**4.4**	3.71	3.74	4.02	0.05
333	3,4,5-Trichlorophenol	000609-19-8	**3.6**	2.39	2.41	3.12	0.02
334	2-Chlorobenzamide	000609-66-5	1.5	1.43	1.77	1.35	0.03
335	2-Nitrobenzamide	000610-15-1	**1.5**	1.23	1.88	1.04	0.05
336	*N*,*N*-Dimethylbenzamide	000611-74-5	**1.5**	1.59	1.71	1.26	0.02
337	2-Aminoanthracene	000613-13-8	**4.5**	3.99	3.86	3.81	0.07
338	*N*-Methylbenzamide	000613-93-4	**1.4**	1.44	1.89	1.50	0.02
339	Ethyl 3,5-dinitrobenzoate	000618-71-3	2.7	1.91	2.17	2.06	0.02
340	3,5-Dinitroaniline	000618-87-1	**2.6**	1.91	2.17	2.06	0.02
341	4-Methylbenzamide	000619-55-6	**1.8**	1.35	1.88	1.58	0.02
342	4-Nitrobenzamide	000619-80-7	**1.9**	1.23	1.88	1.39	0.03
343	3-Bromoacetanilide	000621-38-5	**2**	2.08	1.91	2.10	0.02
344	4-Ethyltoluene	000622-96-8	**3.6**	2.99	2.49	2.48	0.03
345	3,5-Dichloroaniline	000626-43-7	**2.4**	2.51	2.43	2.58	0.02
346	1,1,1,2-Tetrachloroethane	000630-20-6	**1.7**	2.44	2.55	2.07	0.03
347	Tetrachlorophthalate	000632-58-6	**3.3**	2.54	2.90	2.97	0.02
348	1,2,3,4-Tetrachlorobenzene	000634-66-2	**3.8**	3.42	3.34	3.63	0.04
349	2,3,4-Trichloroaniline	000634-67-3	**2.6**	2.94	2.83	2.97	0.02

Table S7. *Cont.*

ID	Chemical Compound	Chemical Abstracts Service Number	Experimental	Predicted Equation (1)	Predicted Equation (2)	Predicted Equation (3)	h_i
350	2,3,4,5-Tetrachloroaniline	000634-83-3	3	3.23	3.23	3.35	0.03
351	1,2,3,5-Tetrachlorobenzene	000634-90-2	**3.4**	3.35	3.34	3.63	0.04
352	3-Nitrobenzamide	000645-09-0	2	1.22	1.52	1.39	0.03
353	Pentafluorophenyl methyl sufone	000651-85-4	**1.5**	2.12	1.63	2.36	0.03
354	2-Fluorophenylurea	000656-31-5	**1.3**	1.20	1.46	1.37	0.02
355	4-Fluorophenylurea	000659-30-3	1.5	1.22	1.46	1.37	0.02
356	Simatone	000673-04-1	**2.3**	2.00	1.37	2.72	0.02
357	Diethylacetamide	000685-91-6	1.8	1.02	1.06	0.78	0.02
358	2,3,5-Trimethylphenol	000697-82-5	**3.6**	1.95	1.66	2.74	0.02
359	Propanil	000709-98-8	**2.2**	2.56	2.69	2.50	0.01
360	Anthracene-9-carboxylic acid	000723-62-6	**2.7**	3.39	3.56	3.74	0.03
361	Phosmet	000732-11-6	**2.9**	2.50	2.63	2.94	0.05
362	Bensulide	000741-58-2	**3.5**	3.17	3.23	3.43	0.02
363	EPTC	000759-94-4	**2.4**	1.73	1.88	2.18	0.03
364	3-Fluorophenylurea	000770-19-4	**1.8**	1.19	1.46	1.37	0.02
365	9-Methylanthracene	000779-02-2	**4.8**	4.53	4.12	4.35	0.06
366	9-Acetylanthracene	000784-04-3	**3.6**	3.54	3.81	3.66	0.03
367	Carbophenothion	000786-19-6	**4.7**	3.77	4.11	4.20	0.03
368	Cyclohexylbenzene	000827-52-1	**4.2**	3.89	2.77	3.18	0.04
369	Ametryn	000834-12-8	2.6	2.71	2.31	3.19	0.03
370	1-Phenylazo-2-naphthalenol	000842-07-9	**3.6**	3.21	2.86	3.25	0.03
371	2,3,4,5-Tetrachloronitrobenzene	000879-39-0	**4.2**	2.92	3.20	3.35	0.02
372	Terbutryn	000886-50-0	**2.9**	2.91	2.47	3.40	0.02
373	3-Phenyl-1-cyclohexylurea	000886-59-9	**2.1**	2.59	2.47	2.74	0.02
374	Demeton-*S*-methyl	000919-86-8	**1.5**	1.50	2.14	1.37	0.02
375	2,3,5-Trichlorophenol	000933-78-8	**3.6**	2.37	2.41	3.06	0.02
376	2-Ethylnaphthalene	000939-27-5	3.8	3.84	3.17	3.22	0.02

Table S7. *Cont.*

ID	Chemical Compound	Chemical Abstracts Service Number	Experimental	Predicted Equation (1)	Predicted Equation (2)	Predicted Equation (3)	h_i
377	Fonofos	000944-22-9	**3.3**	3.37	3.23	3.23	0.02
378	Methidathion	000950-37-8	**2**	1.93	2.58	2.20	0.04
379	Carbophenothion-methyl	000953-17-3	**4.7**	3.44	3.78	3.73	0.02
380	Diphenamid	000957-51-7	**2.1**	3.07	2.89	2.65	0.02
381	Stirofos	000961-11-5	**3.1**	3.15	2.85	3.05	0.02
382	3-Phenyl-1-methylurea	001007-36-9	**1.3**	1.56	1.58	1.54	0.02
383	Heptachlor epoxid	001024-57-3	4	4.12	3.76	4.00	0.04
384	Pebulate	001114-71-2	**2.8**	2.00	2.05	2.42	0.03
385	1-Ethylnaphthalene	001127-76-0	**3.8**	3.85	3.17	3.22	0.02
386	Butyranilide	001129-50-6	**1.7**	2.21	2.05	1.96	0.01
387	Cycloate	001134-23-2	**2.5**	2.21	2.26	2.68	0.03
388	Dichlobenil	001194-65-6	**2.4**	2.22	2.47	2.72	0.02
389	Tetrachlorocatechol	001198-55-6	**1.6**	2.69	2.67	3.22	0.02
390	9-Anthracenemethanol	001468-95-7	3.6	3.33	3.65	3.39	0.03
391	5-Indanol	001470-94-6	**3.6**	2.13	1.72	2.79	0.02
392	Butyl-*N*-phenylcarbamate	001538-74-5	**2.3**	2.41	2.13	2.44	0.02
393	Carbofuran	001563-66-2	**1.6**	2.19	1.94	2.10	0.01
394	Trifluralin	001582-09-8	4.2	3.25	3.94	3.61	0.04
395	Atratone	001610-17-9	**2.6**	2.21	1.53	2.92	0.02
396	Prometon	001610-18-0	**2.6**	2.42	1.70	3.11	0.02
397	*N*,*N*′-Diethylhydrazine	001615-80-1	**1.2**	1.34	0.85	0.60	0.03
398	Aldicarb sulfoxide	001646-87-3	**0.6**	1.10	1.13	0.44	0.04
399	Aldicarb sulfone (=aldoxycarb)	001646-88-4	**0.7**	1.28	0.99	0.50	0.04
400	Bromoxynil	001689-84-5	**2.3**	2.18	1.80	3.19	0.03
401	Bromoxynil octanoate	001689-99-2	**4**	3.72	3.02	4.06	0.04
402	Pyrazon	001698-60-8	**2.1**	2.19	2.66	1.70	0.04
403	Monolinuron	001746-81-2	**2.2**	2.01	2.07	2.00	0.01

Table S7. *Cont.*

ID	Chemical Compound	Chemical Abstracts Service Number	Experimental	Predicted Equation (1)	Predicted Equation (2)	Predicted Equation (3)	h_i
404	Diamidafos	001754-58-1	**1.5**	1.33	1.18	0.91	0.02
405	Nitrofen	001836-75-5	**3.7**	3.75	3.40	3.55	0.02
406	Chlornitrofen	001836-77-7	3.9	4.02	3.80	3.94	0.02
407	Dimethyl 2,3,5,6-Tetrachloroterephthalate	001861-32-1	**3.7**	2.82	3.24	3.17	0.02
408	Benfluralin	001861-40-1	**4**	3.30	3.94	3.61	0.04
409	Chlorothalonil	001897-45-6	**3.2**	2.83	3.37	3.38	0.03
410	Atrazine	001912-24-9	**2.2**	2.46	2.00	2.83	0.02
411	Ipazine	001912-25-0	**3.1**	2.85	2.36	3.35	0.02
412	Trietazine	001912-26-1	**2.8**	2.64	2.19	3.16	0.02
413	Dicamba	001918-00-9	**1.5**	2.11	2.28	2.03	0.01
414	Picloram	001918-02-1	**1.3**	1.49	2.32	1.87	0.03
415	Chlorthiamid	001918-13-4	**2.3**	2.37	3.03	2.78	0.02
416	Propachlor	001918-16-7	**2.4**	2.46	2.52	2.22	0.01
417	Methyl 3,4-dichlorophenylcarbamate	001918-18-9	**2.7**	2.32	2.43	2.52	0.01
418	Vernolate	001929-77-7	**2.4**	1.93	2.05	2.42	0.03
419	Nitrapyrin	001929-82-4	**2.6**	3.03	2.84	2.62	0.02
420	Methyl-*N*-phenylcarbamate	001943-79-9	**1.7**	1.78	1.63	1.74	0.02
421	Chlorbufam	001967-16-4	**2.2**	2.37	1.98	2.51	0.01
422	4-Bromophenylurea	001967-25-5	**2.1**	1.65	1.61	1.96	0.02
423	3-Chlorophenylurea	001967-27-7	**2**	1.55	1.82	1.73	0.02
424	Chloroxuron	001982-47-4	**3.5**	3.40	3.15	3.39	0.02
425	Siduron	001982-49-6	**2.5**	2.80	2.64	2.93	0.02
426	Butylate	002008-41-5	**2.9**	1.98	2.22	2.56	0.04
427	2,6-Dichlorobenzamide	002008-58-4	**0.5**	1.65	2.17	1.55	0.03
428	Methiocarb	002032-65-7	**2.3**	2.01	2.16	2.46	0.01
429	4,4′-Dichlorobiphenyl	002050-68-2	**4.3**	4.10	4.02	4.22	0.05
430	2-Chlorobiphenyl	002051-60-7	**3.5**	3.96	3.62	3.83	0.04

Table S7. *Cont.*

ID	Chemical Compound	Chemical Abstracts Service Number	Experimental	Predicted Equation (1)	Predicted Equation (2)	Predicted Equation (3)	h_i
431	3-Chlorobiphenyl	002051-61-8	**4.4**	3.97	3.62	3.83	0.04
432	2,6-dinitro-*N*-n-propyl-trifluoro-p-toluidine	002077-99-8	**3.6**	2.98	3.43	2.85	0.02
433	EPN	002104-64-5	3.1	3.67	3.28	3.80	0.02
434	Ethyl 1-naphthaleneacetate	002122-70-5	**2.5**	2.93	2.91	3.02	0.02
435	2,3,5,6-Tetrachloroterephthalic acid	002136-79-0	**3.5**	2.53	2.90	2.24	0.02
436	3,4-Dichloroacetanilide	002150-93-8	2.3	2.32	2.52	2.27	0.01
437	Hydroxyatrazine	002163-68-0	**3**	1.68	2.00	2.50	0.03
438	Fluometuron	002164-17-2	**2**	2.19	2.87	1.91	0.02
439	Molinate	002212-67-1	**1.9**	1.70	1.92	2.26	0.02
440	Octachloronaphthalene	002234-13-1	**5.9**	5.56	6.07	6.20	0.12
441	Diallate	002303-16-4	**3.3**	2.43	2.32	2.91	0.03
442	Triallate	002303-17-5	**3.4**	2.73	2.62	3.23	0.03
443	Pentanochlor	002307-68-8	**2.8**	3.02	2.94	3.04	0.02
444	Phosalone	002310-17-0	**3**	3.34	3.72	3.85	0.03
445	Propargite	002312-35-8	3.6	3.50	3.54	3.87	0.04
446	3,4-Dichlorophenylurea	002327-02-8	**2.5**	1.87	2.22	2.13	0.02
447	Mirex	002385-85-5	**6**	6.63	6.13	5.63	0.10
448	Xylicarb	002425-10-7	**1.7**	1.74	1.54	1.70	0.02
449	Benzaloxime-*N*-methylcarbamate	002426-12-2	**1.8**	1.77	1.56	1.75	0.02
450	Oxythioquinox	002439-01-2	**3.4**	2.19	2.20	3.77	0.07
451	Tetrachloroguaiacol	002539-17-5	**2.9**	2.80	2.84	2.97	0.02
452	Etridiazole	002593-15-9	**3**	2.43	2.01	2.65	0.02
453	6-Aminochrysene	002642-98-0	**5.2**	4.93	4.98	4.82	0.12
454	4,5,6-Trichloroguaiacol	002668-24-8	**2.9**	2.49	2.44	2.58	0.02
455	1,4-Dichloro-2,5-dimethoxybenzene	002675-77-6	**3.1**	2.81	2.21	2.59	0.02
456	Trimethacarb	002686-99-9	**2.6**	1.99	1.84	2.11	0.02
457	Chlorpyrifos	002921-88-2	3.9	3.74	3.45	3.80	0.03

Table S7. *Cont.*

ID	Chemical Compound	Chemical Abstracts Service Number	Experimental	Predicted Equation (1)	Predicted Equation (2)	Predicted Equation (3)	h_i
458	Captafol	002939-80-2	**3.3**	2.83	2.88	3.01	0.02
459	Clopidol	002971-90-6	**2.8**	1.87	2.59	1.89	0.02
460	3-Bromophenylurea	002989-98-2	**2.1**	1.63	1.61	1.96	0.02
461	Metobromuron	003060-89-7	**2.1**	2.09	1.86	2.23	0.01
462	Fensulfothion sulfide	003070-15-3	**3.2**	3.57	3.17	3.49	0.02
463	Tetrapropyl dithiopyrophosphate	003244-90-4	**3.8**	3.79	3.22	3.94	0.05
464	Asulam	003337-71-1	**2**	1.14	1.32	1.14	0.04
465	Temephos	003383-96-8	**5**	4.88	4.22	5.02	0.04
466	Pipron	003478-94-2	**3.7**	3.34	3.65	3.57	0.03
467	2,3,5,6-Tetrachloroaniline	003481-20-7	3.9	3.22	3.23	3.35	0.03
468	3-(3,4-Dichlorophenyl)-1-methylurea	003567-62-2	**2.5**	2.10	2.39	2.32	0.01
469	4-Biphenylmethanol	003597-91-9	**2.6**	2.89	2.90	2.75	0.02
470	TEDP	003689-24-5	**2.7**	2.92	2.54	3.00	0.02
471	Fenobucarb	003766-81-2	**1.7**	2.59	2.04	2.23	0.02
472	Metizolin	003813-05-6	**1.5**	2.02	2.10	2.52	0.03
473	Methyl-*N*-(3-chlorophenyl)carbamate	004090-00-0	**2.2**	2.00	2.03	2.13	0.01
474	Dipropetryn	004147-51-7	**3.1**	3.11	2.64	3.61	0.02
475	6-Chloropicolinic acid	004684-94-0	**1.2**	1.42	1.36	1.53	0.02
476	Nitralin	004726-14-1	**2.9**	2.89	2.96	2.88	0.02
477	1-Naphthalenemethanol	004780-79-4	**2.3**	2.40	2.53	2.38	0.02
478	Carboxin	005234-68-4	**2.4**	2.57	2.31	2.10	0.03
479	Oxycarboxin	005259-88-1	**1.7**	2.42	1.91	2.09	0.04
480	3-Chloro-4-methoxyaniline	005345-54-0	**1.9**	2.19	1.93	1.88	0.02
481	Propyl-*N*-phenylcarbamate	005532-90-1	**2.1**	2.16	1.96	2.21	0.02
482	Chlorpyrifos methyl	005598-13-0	**3.5**	3.39	3.12	3.33	0.02
483	Terbacil	005902-51-2	**1.7**	1.55	2.32	1.87	0.02
484	Terbuthylazine	005915-41-3	**2.3**	2.66	2.21	3.04	0.02

Table S7. *Cont.*

ID	Chemical Compound	Chemical Abstracts Service Number	Experimental	Predicted Equation (1)	Predicted Equation (2)	Predicted Equation (3)	h_i
485	Bis(2-ethylhexyl) terephthalate	006422-86-2	**4.2**	4.90	4.40	4.82	0.18
486	3,5,6-trichloro-2-pyridinol	006515-38-4	**2.1**	2.09	2.55	1.92	0.06
487	Monocrotophos	006923-22-4	**0**	0.85	0.44	0.54	0.03
488	3-Methyl-4-bromoaniline	006933-10-4	**2.3**	2.50	1.98	2.67	0.02
489	2,4,4′-Trichlorobiphenyl	007012-37-5	**4.6**	4.30	4.42	4.61	0.06
490	3-Chloro-4-methoxyacetanilide	007073-42-9	**2**	2.09	2.14	1.93	0.02
491	Mecoprop	007085-19-0	1.3	2.30	2.06	2.34	0.02
492	Pyroxychlor	007159-34-4	**3.5**	3.03	2.48	2.89	0.02
493	3-(4-Methylphenyl)-1,1-dimethylurea	007160-01-2	**1.5**	1.85	1.91	1.89	0.01
494	3-(4-Methoxyphenyl)-1,1-dimethylurea	007160-02-3	**1.4**	1.76	1.78	1.67	0.02
495	Chloramben methyl	007286-84-2	2.7	1.88	2.45	2.36	0.01
496	Prometryn	007287-19-6	**2.9**	2.92	2.47	3.38	0.02
497	Crotoxyphos(trans)	007700-17-6	**2**	2.56	1.77	2.30	0.02
498	Mevinphos(cis)	007786-34-7	**2**	1.06	0.48	0.91	0.02
499	Toxaphene	008001-35-2	**3.1**	5.06	4.45	4.42	0.07
500	trans-1,3-Dichloropropene	010061-02-6	**1.4**	2.00	1.37	1.72	0.03
501	Methamidophos	010265-92-6	**0.7**	0.37	0.63	0.25	0.04
502	Terbufos sulfoxide	010548-10-4	**2.2**	2.46	3.00	2.27	0.01
503	Carbendazim (MBC)	010605-21-7	**2.4**	1.93	2.26	2.19	0.04
504	Ancymidol	012771-68-5	**2.1**	2.21	2.38	2.28	0.02
505	2,2′-Dichlorobiphenyl	013029-08-8	**3.9**	4.12	4.02	4.22	0.05
506	Terbufos	013071-79-9	**2.8**	3.20	3.27	3.27	0.02
507	3-Trifluoromethylphenylurea	013114-87-9	**2**	1.73	2.53	1.63	0.02
508	3-Phenyl-1-cyclopropylurea	013140-86-8	**1.7**	1.84	1.96	2.04	0.02
509	3-Phenyl-1-cyclopentylurea	013140-89-1	1.9	2.35	2.30	2.51	0.01
510	Phosphamidon	013171-21-6	0.9	1.91	1.25	1.42	0.02
511	Ethoprophos	013194-48-4	**1.8**	2.05	2.57	2.39	0.02

Table S7. *Cont.*

ID	Chemical Compound	Chemical Abstracts Service Number	Experimental	Predicted Equation (1)	Predicted Equation (2)	Predicted Equation (3)	h_i
512	Chlorbromuron	013360-45-7	**2.7**	2.39	2.27	2.62	0.01
513	Desmedipham	013684-56-5	**3.2**	3.39	2.89	2.96	0.02
514	Phenmedipham	013684-63-4	3.4	3.31	2.88	2.99	0.02
515	Fensulfothion sulfone	014255-72-2	**2.2**	2.58	2.69	2.54	0.02
516	Napropamide	015299-99-7	**2.7**	3.38	3.34	2.95	0.02
517	Chlorotoluron	015545-48-9	2	2.08	2.31	2.28	0.01
518	2,2′,6,6′-Tetrachlorobiphenyl	015968-05-5	**5**	4.61	4.82	5.01	0.07
519	Alachlor	015972-60-8	**2.3**	2.86	2.91	2.70	0.02
520	Methomyl	016752-77-5	**1.8**	1.09	1.06	1.10	0.02
521	Benomyl	017804-35-2	2.7	2.74	2.26	2.83	0.05
522	Methyl-*N*-(3,4-dichlorophenyl)carbamate	018315-50-9	**2.7**	2.32	2.43	2.52	0.01
523	Methabenzthiazuron	018691-97-9	2.8	2.18	2.07	2.81	0.03
524	Chlorfenvinphos(trans)	018708-86-6	**2.5**	3.16	2.78	3.13	0.02
525	Chlorfenvinphos(cis)	018708-87-7	2.5	3.16	2.78	3.13	0.02
526	Oryzalin	019044-88-3	**3.1**	2.26	2.51	2.78	0.02
527	Oxadiazon	019666-30-9	**3.5**	3.62	3.48	3.53	0.03
528	Metoxuron	019937-59-8	**1.7**	2.03	2.18	2.07	0.01
529	Methazole	020354-26-1	**3.5**	2.44	2.26	2.82	0.01
530	3-(3-Chloro-4-methoxylphenyl)-1-methylurea	020782-57-4	**1.8**	1.87	2.01	1.98	0.02
531	3-(3-Chlorophenyl)-1-methylurea	020940-42-5	**1.9**	1.78	1.99	1.93	0.02
532	Metribuzin	021087-64-9	**1.9**	1.42	2.05	1.77	0.02
533	Leptophos	021609-90-5	**4.2**	4.70	4.35	4.88	0.04
534	Cyanazine	021725-46-2	**2.3**	2.14	1.95	2.76	0.03
535	3-(3-Chloro-4-methylphenyl)-1-methylurea	022175-22-0	**2.1**	1.92	2.14	2.19	0.01
536	Fenamiphos	022224-92-6	2.5	2.31	2.45	2.59	0.02
537	Bendiocarb	022781-23-3	**2.8**	2.07	1.65	2.23	0.01
538	Pirimicarb(=Pirimor)	023103-98-2	**2.1**	1.85	1.60	1.88	0.02

Table S7. *Cont.*

ID	Chemical Compound	Chemical Abstracts Service Number	Experimental	Predicted Equation (1)	Predicted Equation (2)	Predicted Equation (3)	h_i
539	Oxamyl	023135-22-0	1	0.88	1.15	0.38	0.06
540	Butachlor	023184-66-9	**3.1**	3.54	3.42	3.40	0.03
541	Thiophanate-methyl	023564-05-8	**3.3**	2.33	2.78	2.39	0.09
542	Pronamide	023950-58-5	2.3	2.38	2.46	2.87	0.02
543	Piperophos	024151-93-7	3.4	3.04	3.11	3.23	0.02
544	Bentazon	025057-89-0	**1.5**	1.68	1.88	2.02	0.02
545	3-Chloro-4-methoxyphenylurea	025277-05-8	2	1.64	1.84	1.79	0.02
546	Isofenphos	025311-71-1	**2.8**	3.04	2.83	3.23	0.02
547	Iprobenfos	026087-47-8	**2.4**	2.66	3.03	2.76	0.02
548	Ethofumesate	026225-79-6	**2.1**	2.50	2.44	2.23	0.02
549	Secbumeton	026259-45-0	**2.8**	2.49	1.70	3.15	0.02
550	Chlornidine	026389-78-6	**3.9**	2.79	3.24	3.22	0.02
551	Profluralin	026399-36-0	**4**	3.30	4.15	3.88	0.04
552	Triforine	026644-46-2	2.8	2.20	3.09	2.29	0.02
553	Norflurazon	027314-13-2	**3.3**	2.90	3.95	2.18	0.02
554	3-(3-Methoxyphenyl)-1,1-dimethylurea	028170-54-9	**1.7**	1.74	1.78	1.67	0.02
555	Thiobencarb	028249-77-6	**3.3**	2.50	2.90	3.06	0.02
556	Dinitramine	029091-05-2	**3.6**	3.11	3.66	3.13	0.02
557	Pirimiphos-methyl	029232-93-7	**3**	3.11	2.11	3.39	0.02
558	3-Chloro-4-bromonitrobenzene	029682-39-1	**2.6**	2.42	2.19	2.80	0.01
559	Acephate	030560-19-1	0.4	0.71	0.85	0.28	0.04
560	Fenamiphos Sulfoxide	031972-43-7	1.6	1.84	1.94	1.58	0.02
561	Fenamiphos Sulfone	031972-44-8	**1.6**	2.12	1.97	1.65	0.02
562	2,4-DB butoxyethyl ester	032357-46-3	**2.7**	3.43	3.40	3.40	0.04
563	2,3′,4′,5-Tetrachlorobiphenyl	032598-11-1	**4.9**	4.61	4.82	5.01	0.07
564	Fluchloralin	033245-39-5	**3.6**	3.19	4.07	3.58	0.03
565	Butralin	033629-47-9	**4**	3.71	3.01	3.67	0.03

Table S7. *Cont.*

ID	Chemical Compound	Chemical Abstracts Service Number	Experimental	Predicted Equation (1)	Predicted Equation (2)	Predicted Equation (3)	h_i
566	Isopropalin	033820-53-0	**4**	3.51	3.31	4.02	0.04
567	Tebuthiuron	034014-18-1	**1.8**	1.89	2.24	1.98	0.02
568	Isoproturon	034123-59-6	**2.1**	2.46	2.24	2.32	0.02
569	Acetochlor	034256-82-1	2.2	2.80	2.91	2.70	0.02
570	2,4′-Dichlorobiphenyl	034883-43-7	**4.1**	4.08	4.02	4.22	0.05
571	2,2′,4,4′,5,5′-Hexachlorobiphenyl	035065-27-1	**5.9**	5.20	5.63	5.78	0.10
572	Diflubenzuron	035367-38-5	**3.8**	3.08	3.10	3.10	0.02
573	Sulprofos	035400-43-2	**4.3**	4.08	3.83	4.22	0.03
574	Imazalil	035554-44-0	**3.7**	3.46	2.81	3.23	0.02
575	2,2′,5,5′-Tetrachlorobiphenyl	035693-99-3	**4.8**	4.58	4.82	5.01	0.07
576	Hexabromobiphenyl	036355-01-8	**4.9**	5.61	4.41	7.12	0.16
577	3-(3,5-Dimethylphenyl)-1,1-dimethylurea	036627-56-2	**1.7**	1.92	2.67	2.16	0.01
578	Iprodione	036734-19-7	**2.2**	2.57	3.33	2.93	0.03
579	2,5,2′-Trichlorobiphenyl	037680-65-2	**4.2**	4.35	4.42	4.61	0.06
580	2,4,2′-Trichlorobiphenyl	037680-66-3	4.8	4.34	4.42	4.61	0.06
581	2,2′,4,5,5′-Pentachlorobiphenyl	037680-73-2	**4.6**	4.89	5.23	5.39	0.09
582	2,2′,3,4,5′-Pentachlorobiphenyl	038380-02-8	**4.7**	4.94	5.23	5.39	0.09
583	2,2′,3,3′,4,4′-Hexachlorobiphenyl	038380-07-3	5.7	5.30	5.63	5.78	0.10
584	Diethatyl-Ethyl	038727-55-8	3.1	3.01	3.13	2.83	0.02
585	Pendimethalin	040487-42-1	**3**	3.29	2.96	3.53	0.03
586	Profenofos	041198-08-7	**3**	3.20	3.25	3.64	0.02
587	Metamitron	041394-05-2	**2.2**	1.73	2.46	1.93	0.03
588	Tricyclazole	041814-78-2	**3.1**	2.96	2.72	3.14	0.06
589	Isazophos	042509-80-8	**2**	3.22	2.90	2.85	0.02
590	Bifenox	042576-02-3	**3.7**	3.60	3.54	3.49	0.02
591	Oxyfluorofen	042874-03-3	**4.9**	4.16	4.31	3.73	0.03
592	Triadimefon	043121-43-3	**2.6**	2.86	3.02	2.58	0.01

Table S7. *Cont.*

ID	Chemical Compound	Chemical Abstracts Service Number	Experimental	Predicted Equation (1)	Predicted Equation (2)	Predicted Equation (3)	h_i
593	Metolachlor	051218-45-2	**2.5**	2.85	3.08	2.63	0.02
594	Hexazinone	051235-04-2	1.7	1.84	1.96	2.17	0.02
595	Diclofop-Methyl	051338-27-3	**4.2**	4.03	3.48	3.51	0.02
596	Fenvalerate	051630-58-1	**4.4**	5.12	4.60	4.93	0.04
597	Thidiazuron	051707-55-2	**2**	2.21	2.31	2.41	0.03
598	Cypermethrin	052315-07-8	**4.6**	4.71	4.04	4.64	0.04
599	Permethrin	052645-53-1	**4**	4.68	4.09	5.07	0.06
600	2,2′,3,4,5,5′-Hexachlorobiphenyl	052712-04-6	**6**	5.23	5.63	5.78	0.10
601	2,2′,3,4,5,5′,6-Heptachlorobiphenyl	052712-05-7	**5.8**	5.56	6.03	6.18	0.12
602	Ethalfluralin	055283-68-6	**3.8**	3.05	3.60	3.66	0.03
603	Dimethipin	055290-64-7	**0.5**	1.53	1.18	1.44	0.03
604	Triclopyr	055335-06-3	**1.6**	2.22	3.11	2.30	0.01
605	Isouron	055861-78-4	**2.5**	1.89	1.95	1.55	0.02
606	Terbufos sulfone	056070-16-7	**2.2**	2.68	2.86	2.33	0.01
607	Metalaxyl	057837-19-1	**1.6**	2.25	2.57	1.84	0.02
608	Tridiphane	058138-08-2	**3.8**	3.57	3.67	3.71	0.03
609	Thiodicarb	059669-26-0	**2.5**	2.23	2.31	2.05	0.02
610	Fluridone	059756-60-4	**2.8**	4.03	5.17	3.56	0.02
611	Fenarimol	060168-88-9	**3**	3.29	3.66	3.47	0.03
612	Propiconazole	060207-90-1	3.4	3.62	3.70	3.28	0.02
613	Fluorochloridone	061213-25-0	**2.6**	3.11	3.99	2.76	0.02
614	Flumetralin	062924-70-3	**4**	4.03	5.10	4.37	0.04
615	Pentyl-*N*-phenylcarbamate	063075-06-9	2.6	2.65	2.30	2.68	0.02
616	Chlorosulfuron	064902-72-3	**1.6**	2.56	2.06	2.80	0.05
617	Cyromazine	066215-27-8	**2.3**	1.43	1.40	2.41	0.11
618	Esfenvalerate	066230-04-4	**3.7**	5.12	4.60	4.93	0.04
619	Fenoxaprop-Ethyl	066441-23-4	**4**	4.29	3.29	3.93	0.02

Table S7. *Cont.*

ID	Chemical Compound	Chemical Abstracts Service Number	Experimental	Predicted Equation (1)	Predicted Equation (2)	Predicted Equation (3)	h_i
620	Tralomethrin	066841-25-6	**5**	5.64	4.90	5.66	0.06
621	Prochloraz	067747-09-5	**2.7**	3.30	3.52	3.54	0.02
622	Cyfluthrin	068359-37-5	**5**	4.71	4.08	4.27	0.03
623	Fluazifop-butyl	069806-50-4	**2.8**	4.32	4.05	3.54	0.05
624	Flucythrinate	070124-77-5	**5**	5.25	4.87	4.66	0.04
625	4-Chlorobenzaloxime-*N*-methylcarbamate	071059-53-5	2	1.95	1.96	2.14	0.01
626	Sethoxydim	074051-80-2	**2**	3.02	2.72	2.60	0.02
627	Sulfometuron-Methyl	074222-97-2	1.8	2.64	2.23	2.49	0.05
628	Metasulfron methyl	074223-64-6	**1.7**	2.37	1.91	2.62	0.05
629	Quizalofop-ethyl	076578-14-8	**2.7**	4.27	3.45	3.76	0.02
630	Flutriafol	076674-21-0	**1.9**	2.42	3.24	2.54	0.02
631	3-(3,5-Dimethyl-4-bromophenyl)-1,1-dimethylurea	078508-43-7	**2.5**	2.28	2.26	2.77	0.01
632	4-Phenoxylphenylurea	078508-44-8	**2.6**	2.76	2.41	2.72	0.02
633	3-Methyl-4-fluorophenylurea	078508-45-9	**1.8**	1.31	1.61	1.63	0.02
634	3-Methyl-4-bromophenylurea	078508-46-0	**2.4**	1.77	1.77	2.22	0.02
635	Hexythiazox	078587-05-0	**3.8**	3.46	3.67	4.23	0.03
636	Fenoxycarb	079127-80-3	**3**	3.36	2.80	3.30	0.02
637	Thiameturon-methyl	079277-27-3	1.7	2.21	1.70	2.26	0.06
638	Imazapyr acid	081334-34-1	**2**	1.85	1.98	2.00	0.02
639	Isoxaben	082558-50-7	**2.4**	3.78	3.41	3.02	0.02
640	Bifenthrin	082657-04-3	5.4	5.29	5.86	5.30	0.09
641	Cinmethylin	087818-31-3	**2.6**	3.97	3.27	3.17	0.03
642	Chlorimuron ethyl	090982-32-4	2	2.86	2.45	2.85	0.05
643	Cyhalothrin	091465-08-6	**5.3**	5.07	4.14	4.61	0.06

Table S8. List of mathematical equations used in present study.

Equations List	No.
$S = \sqrt{\frac{\sum_{i=1}^{N}\left(p_i^{exp} - p_i^{pred}\right)^2}{N - d - 1}}$	1
$RMS = \sqrt{\frac{\sum_{i=1}^{N}\left(p_i^{exp} - p_i^{pred}\right)^2}{N}}$	2
$h_i = x_i(\mathbf{X}^T\mathbf{X})^{-1}x_i^T$	3
$h^* = 3(d + 1)/N_{train}$	4
$k = \frac{\sum_{i=1}^{N_{test}}\left(p_i^{exp} p_i^{pred}\right)^2}{\sum_{i=1}^{N_{test}}\left(p_i^{pred}\right)^2}$	5
$k^{'} = \frac{\sum_{i=1}^{N_{test}}\left(p_i^{exp} p_i^{pred}\right)^2}{\sum_{i=1}^{N_{test}}\left(p_i^{\mathrm{exp}}\right)^2}$	6
$p_0^{exp} = kp^{pred}$	7
$p_0^{pred} = k^{'}p^{exp}$	8
$R_0^2 = 1 - \frac{\sum_{i=1}^{N_{test}}\left(p_i^{exp} - p_{0i}^{exp}\right)^2}{\sum_{i=1}^{N_{test}}\left(p_i^{exp} - p_{av}^{exp}\right)^2}$	9
$R_0^{'2} = 1 - \frac{\sum_{i=1}^{N_{test}}\left(p_i^{pred} - p_{0i}^{pred}\right)^2}{\sum_{i=1}^{N_{test}}\left(p_i^{pred} - p_{av}^{pred}\right)^2}$	10

Table S8. *Cont.*

Equations List	No.
$R_m^2 = R_{test}^2\left(1 - \left\|\sqrt{R_{test}^2 - R_0^2}\right\|\right)$	11
${}^{k+1}EC_j = \sum_{a_{ij}\neq 0} CW({}^{k}EC_i)$	12
$DCW = \sum_j CW(\mathrm{SA}_j)$	13

S: standard deviation; *N*: number of molecules; p_i^{exp}: experimental activity for compound *i*; p_i^{pred}: predicted activity for compound *i*; *d*: number of descriptors; *RMS* = root mean square deviation; h_i: leverage for compound *i*; x_i: descriptor vector for *i*; $\mathbf{X}$: model matrix for the training set (train); N_{train} : number of molecules in train; N_{test} = number of molecules in test; h^* : warning leverage; p_{0i}^{exp} and p_{0i}^{pred} : calculated activities for *i* in test set in regressions through the origin of p^{exp} against p^{pred} and p^{pred} against p^{exp}, respectively; R_0^2 and $R_0^{'2}$: squared correlation coefficients for regressions through the origin in test set of p^{exp} against p^{pred} and p^{pred} against p^{exp}, respectively; p_{av}^{pred}: average value for p^{pred} in test set; p_{av}^{exp}: average value for p^{exp} in test set; R_m^2: modified squared correlation coefficient; R_{test}^2: squared correlation coefficient between observed and predicted values for the test set; *CW*: correlation weight; ${}^{k}EC_i$ Morgan's extended connectivity index of *k*th order for vertex number *i*; a_{ij} : Adjacency matrix element; *DCW*: optimal descriptor; SA: structural attribute.

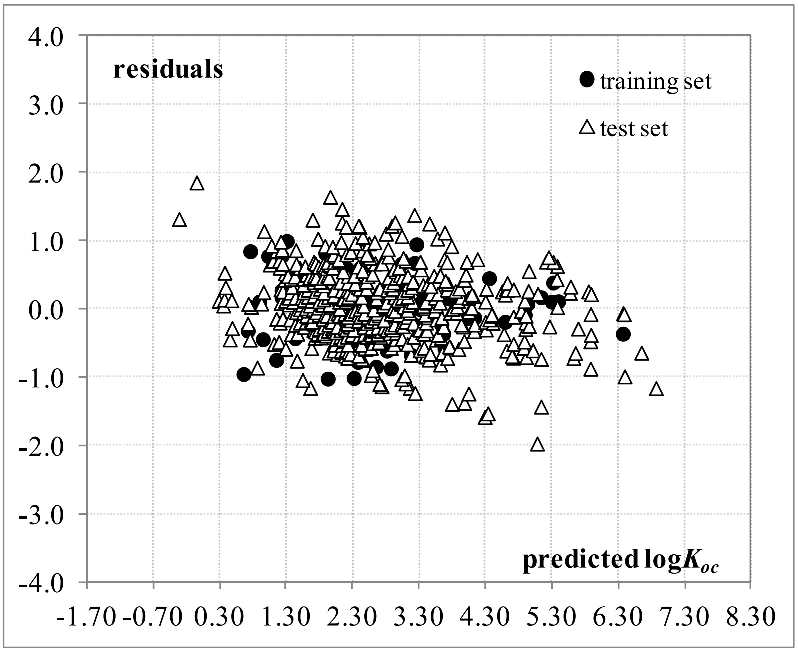

Figure S1. Dispersion plot of residuals for Equation (1).

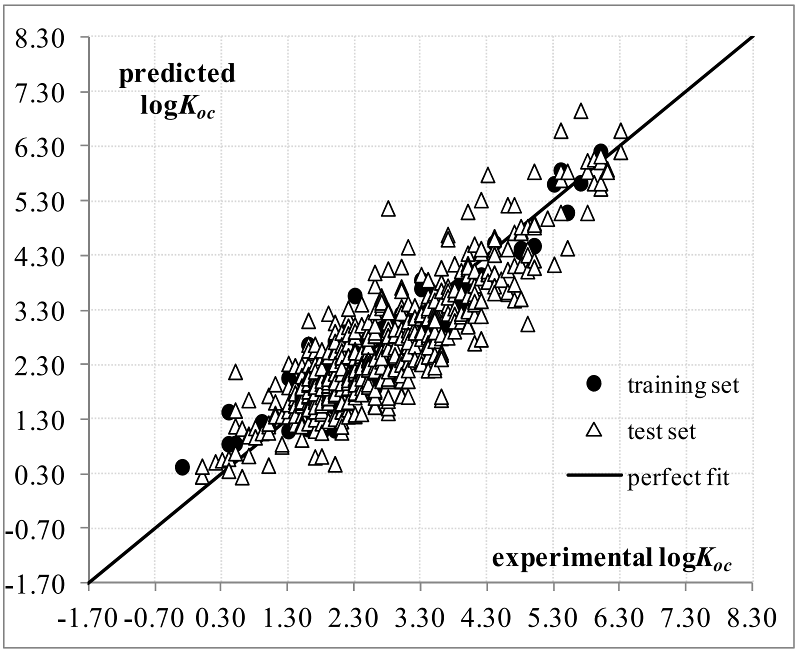

Figure S2. Predicted and experimental $\log K_{oc}$ values according to QSPR based on Equation (2).

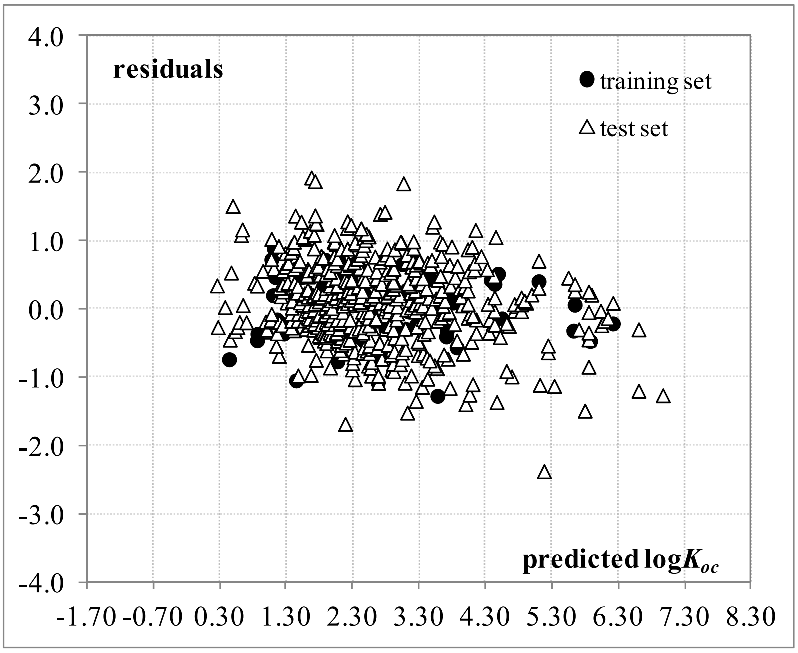

Figure S3. Dispersion plot of residuals for Equation (2).

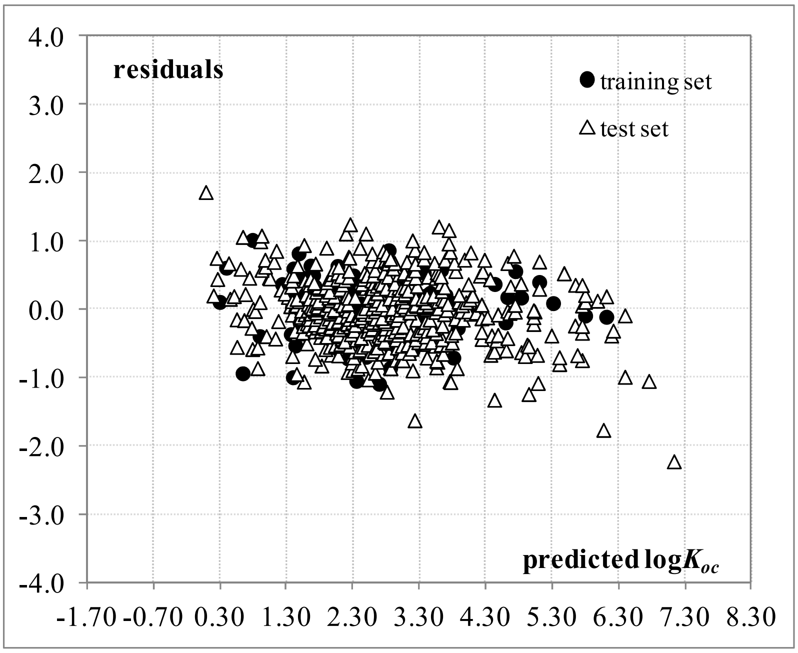

Figure S4. Dispersion plot of residuals for Equation (3).
